# Deploying wearable sensors for pandemic mitigation: A counterfactual modelling study of Canada’s second COVID-19 wave

**DOI:** 10.1371/journal.pdig.0000100

**Published:** 2022-09-06

**Authors:** Nathan Duarte, Rahul K. Arora, Graham Bennett, Meng Wang, Michael P. Snyder, Jeremy R. Cooperstock, Caroline E. Wagner

**Affiliations:** 1 Department of Electrical and Computer Engineering, Faculty of Engineering, McGill University, Montreal, Canada; 2 Department of Community Health Sciences, University of Calgary, Calgary, Canada; 3 Institute of Biomedical Engineering, University of Oxford, Oxford, United Kingdom; 4 Department of Economics, Faculty of Arts, McGill University, Montreal, Canada; 5 Department of Genetics, Stanford University School of Medicine, Stanford University, California, United States of America; 6 Department of Bioengineering, Faculty of Engineering, McGill University, Montreal, Canada; University of Cagliari: Universita degli Studi Di Cagliari, ITALY

## Abstract

Wearable sensors can continuously and passively detect potential respiratory infections before or absent symptoms. However, the population-level impact of deploying these devices during pandemics is unclear. We built a compartmental model of Canada’s second COVID-19 wave and simulated wearable sensor deployment scenarios, systematically varying detection algorithm accuracy, uptake, and adherence. With current detection algorithms and 4% uptake, we observed a 16% reduction in the second wave burden of infection; however, 22% of this reduction was attributed to incorrectly quarantining uninfected device users. Improving detection specificity and offering confirmatory rapid tests each minimized unnecessary quarantines and lab-based tests. With a sufficiently low false positive rate, increasing uptake and adherence became effective strategies for scaling averted infections. We concluded that wearable sensors capable of detecting presymptomatic or asymptomatic infections have potential to help reduce the burden of infection during a pandemic; in the case of COVID-19, technology improvements or supporting measures are required to keep social and resource costs sustainable.

## Author summary

Find-Test-Trace-Isolate (FTTI) systems reliant on lab-based tests are important components of pandemic mitigation but can miss infectious individuals that do not have symptoms and may be limited by slow test result turnaround times. Wearable sensors show promise in continuous, passive detection of respiratory infections, before or absent symptoms. Here, we used a mathematical model to study the counterfactual impact of deploying wearable sensors to detect SARS-CoV-2 infections during Canada’s second COVID-19 wave. We observed a meaningful reduction in the burden of infection but also found that false positive alerts resulting from imperfect detection specificity resulted in high social and resource costs. Improving detection specificity and offering rapid antigen tests to confirm positive alerts both helped minimize unnecessary quarantines and lab-based tests. We found that once the false positive rate was sufficiently reduced, increasing uptake and adherence became effective strategies to scale the number of averted infections. Our study demonstrates that wearable sensors capable of detecting infections before or absent symptoms are promising pandemic mitigation tools. It also provides intuition around how detection performance, uptake, adherence, and supporting policies might shape the impact of broad scale wearable sensor deployment.

## Introduction

Infectious disease outbreaks can have devastating health and economic consequences. Effective public health strategies are crucial for limiting transmission and minimizing these harms. One approach to controlling viral spread during pandemics–a “Find, Test, Trace, Isolate” (FTTI) strategy–relies on identifying and isolating infectious individuals [1]. However, the COVID-19 pandemic has demonstrated that FTTI systems reliant on lab-based tests are often limited by missed hidden infection chains resulting from presymptomatic and asymptomatic transmission, and by slow test result turnaround times [2,3]. Digital contact tracing and rapid testing programs have potential to fill these gaps, but both approaches have faced numerous implementation barriers: inadequate participation levels, concerns around privacy and feasibility, and limited test availability [4–6].

Wearable sensors have already been established as tools to detect deviations from users’ physiological baselines [7]. Recent findings suggest that wearable sensors may also be able to detect infections caused by respiratory pathogens such as SARS-CoV-2, before or absent symptoms [8–10]. Alavi *et al*, for example, developed an algorithm that analyzes patterns in smartwatch-captured overnight resting heart rate and provides real-time alerts of potential presymptomatic and asymptomatic SARS-CoV-2 infection [10]. If such algorithms were widely deployed, wearable sensors could be promising tools for pandemic mitigation; they could help FTTI systems more rapidly identify (and subsequently isolate) infectious individuals, including those without symptoms. Wearable sensors would also offer the unique benefit of passive monitoring, which minimizes required user engagement, and could operate in privacy-preserving fashion because sensor data would not need to be shared with a centralized database. With these potential benefits in mind, several studies have focused on developing wearable sensor-based infectious disease detection algorithms or even using these devices for infectious disease surveillance [8–12]. However, to the best of our knowledge, the potential population-level impact of deploying these devices for pandemic mitigation has yet to be explored.

In this study, we investigated the potential for wearable sensors capable of detecting presymptomatic and asymptomatic infections to help reduce the burden of infection during the acute phase of a pandemic. To do so, we used COVID-19 as an example and explored counterfactual scenarios in which these devices were deployed to combat Canada’s second wave. We built a compartmental epidemiological model in which wearable devices notify users of potential infection and prompt them to seek a confirmatory lab-based test, quarantining while waiting for the result. We aimed to (1) assess the baseline impact of deploying currently available detection algorithms during Canada’s second COVID-19 wave, (2) investigate how detection accuracy and behavioural parameters influence this impact, and (3) explore a complementary strategy wherein rapid antigen tests are used to confirm wearable-based notifications of potential infection.

## Methods

### Counterfactual scenarios

We simulated Canada’s second COVID-19 wave (September 1, 2020 to February 20, 2021). This time window allowed us to capture the dynamics of wearable sensor deployment during an acute phase of the pandemic and at a time when the technology would have been ready and deployable. Further, it allowed us to consider scenarios prior to broad vaccine availability and before then-emerging variants of concern (VOCs) were dominant [13]. Potential reinfections were also likely to be negligible in this timeframe [14]. Although we do not explicitly model vaccines, VOCs, and reinfections, we later consider various hypotheses concerning their potential impact on wearable sensor deployment in the Discussion section.

We first explored a baseline scenario in which wearable device users can download an application with currently available detection algorithms [10]. We then investigated the impact of technology and behavioural parameters: detection sensitivity and specificity; uptake, defined as the proportion of the population that has downloaded the application and uses their wearable device often enough; and adherence, defined as the proportion of users who comply with all recommended next steps after a positive notification. Finally, we considered a complementary intervention wherein users with a positive notification are offered a confirmatory rapid antigen test before they are prompted to seek a lab-based test and quarantine.

### Model description

**Fig 1:**
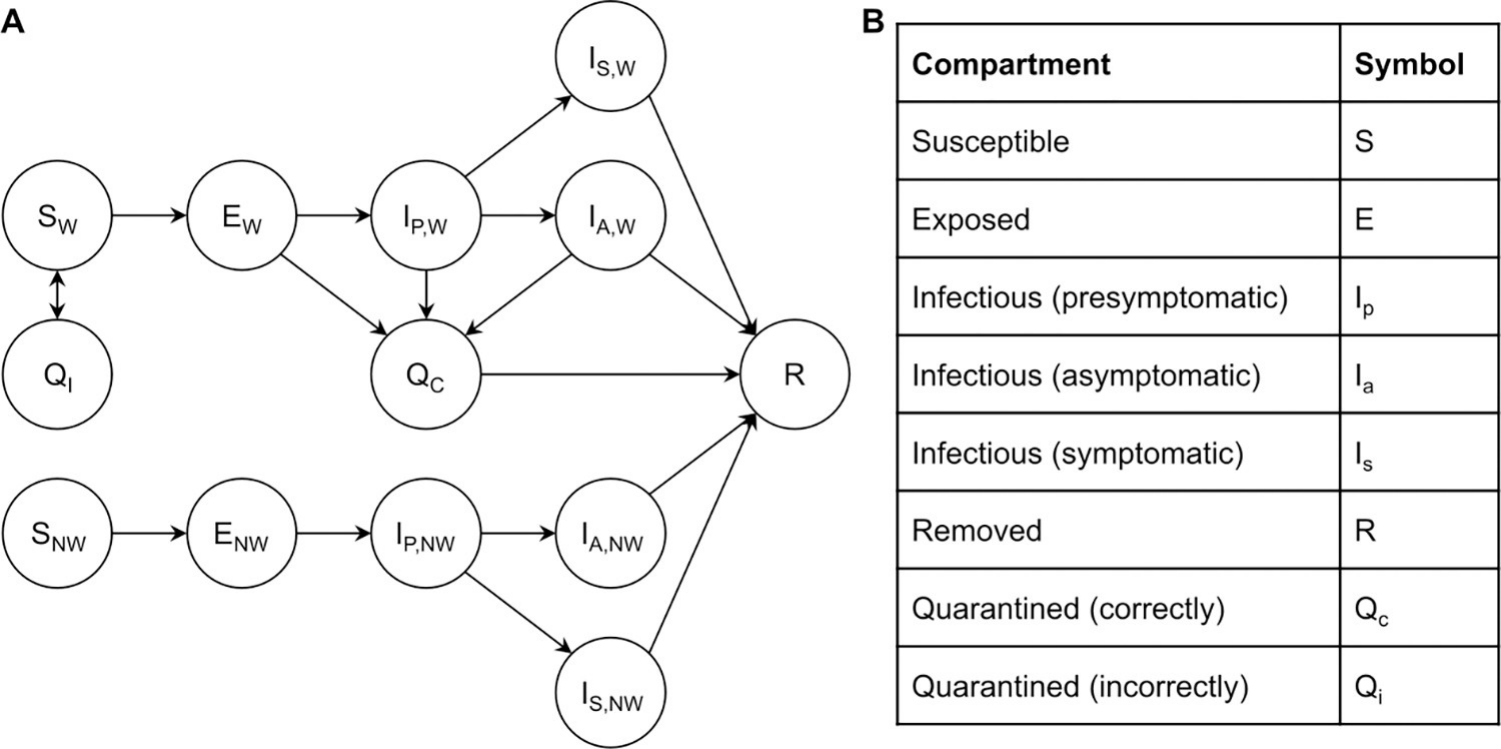
Compartmental model structure. Subscript “W” denotes a wearable user and “NW” denotes otherwise. Two core model equations are presented below; remaining parameters, equations, and assumptions are outlined in Section 1 in S1 Text.

We built a compartmental model based on a *Susceptible*, *Exposed*, *Infectious*, *Removed* (*SEIR*) framework (Fig 1). We split the *Infectious* state into three sub-states: *Presymptomatic*, *Asymptomatic*, and *Symptomatic*. All infected individuals enter the *Presymptomatic* infectious state after a latent period following exposure; some go on to develop symptoms (*Symptomatic*) while others do not (*Asymptomatic*).

To incorporate wearable sensor deployment, we stratified *Susceptible*, *Exposed*, and *Infectious* states by whether individuals are device users or not. Wearable device users can enter *Quarantined* states if they are notified of potential infection, and if they adhere to this notification by seeking a confirmatory lab-based test and quarantining while awaiting the result. We modelled adherence as the fraction of notified users notified who comply with all recommended next steps; accordingly non-adherent users ignore the notification entirely in this framework. We captured adherence in one parameter to preserve model parsimony and considered all values of this parameter (i.e., from 0% to 100%) recognizing the reality there will be great variation in the extent to which notified users are adherent. To explore the notion that adherence may not be “all or nothing” in practice, we separately considered the possibility that non-adherent users who do not take any recommended next steps still act more cautiously (e.g., limiting contacts, wearing a more protective mask) due to the notification (Fig C in S1 Text).

We set the nominal lab-based test turnaround time to 2 days, assumed perfect lab-based test accuracy, and separately explored faster and slower turnaround times (Fig G in S1 Text) [15]. *Susceptible* wearable device users could be *Incorrectly Quarantined* due to a false positive notification and would re-enter the *Susceptible* state after receiving their lab-based test result. *Exposed* and *Infectious* device users would be *Correctly Quarantined* and would enter the *Removed* state (a longer period of isolation until recovery) after their lab-based test confirms infection.

Multiple studies have established the potential for wearable sensors to detect presymptomatic, asymptomatic, and symptomatic SARS-CoV-2 infections [16–19]. With this said, a meaningful yet unknown fraction of *Symptomatic* individuals would have already undergone some degree of quarantining–behaviour already accounted for in the historical transmission rate (β). For this reason, we did not include a pathway for *Symptomatic* device users to enter *Quarantined* states. We separately explored the impact of smaller and larger values for the prevalence of infected individuals that remain asymptomatic (Fig D in S1 Text).

In some scenarios, we also included a step where compliant users take a confirmatory rapid antigen test. If positive, we assumed they then take a lab-based test, quarantining while awaiting the result; if negative, we assumed they return to historical behaviour. In Section 4 in S1 Text, we investigated the impact of rapid antigen test sensitivity (Fig F in S1 Text).

### Simulation approach

To perform simulations, we first extracted the historical transmission rate (β) from the incidence of infection (π) according to Eq (1). In Eq (1), *N* represents the size of the entire population and λ represents the transmission potential of infected individuals without symptoms relative to those with symptoms. Using the true incidence of infection, rather than a time series of incompletely-ascertained cases, is crucial to appropriately capture the extent of historical viral spread [20]. Because estimating π is challenging and was not itself an objective of the present work, we drew from the Institute for Health Metrics and Evaluation (IHME) infection model, a time series nowcasting model that is widely used to understand the historical extent of infection [21–23]. The IHME model estimates π from confirmed cases, hospitalizations, and deaths, and validates results against seroprevalence data. We downloaded these data from the IHME website on December 7, 2021. To ensure our findings were robust to the underlying infection model, we replicated core analyses using estimates of π from the Imperial College London (ICL) infection model (Fig B in S1 Text) [24].


$$\beta =\frac{\pi N}{S(\lambda I_{p}+\lambda I_{a}+I_{s})}$$ (1)

$$\pi =\frac{\beta }{N}[\lambda (aI_{p,w}+I_{p,nw}+aI_{a,w}+I_{a,nw})+(I_{s,w}+I_{s,nw})][{aS}_{w}+S_{nw}]$$ (2)

Next, we applied β according to Eq (2) to simulate counterfactual scenarios. The time series for β that results from Eq (1) incorporates all historical policy measures (e.g., restrictions, business closures, testing availability) and behaviour (e.g., adherence to restrictions, quarantines) that occurred. However, because some *Susceptible*, *Exposed*, and *Infectious* device users now quarantine in simulations, the counterfactual incidence of infection–the π obtained from Eq (2)–decreases relative to historical levels. In Section 4 in S1 Text, we investigated the possibility that device users who are not notified of potential infection act in a riskier fashion (e.g., increasing contacts) relative to historical behaviour (Fig C in S1 Text) [25]. *a* is a coefficient used to study this possibility and is nominally set to 1. When *a* is above 1, the average device user in the *Susceptible*, *Presymptomatic Infectious*, and *Asymptomatic Infectious* compartments acts in a riskier fashion relative to historical behaviour; when *a* is below 1, the average user in these groups acts more cautiously.

We modeled asymptomatic prevalence (ρ), detection algorithm sensitivity (σ_w_) and specificity (ν_w_), and adherence (ψ) as beta-distributed random variables because these parameters were important sources of variance in our assessment of wearable sensors as pandemic mitigation tools. We sampled these variables and used the resulting values to generate an epidemic trajectory. We repeated this process 5,000 times, using these Monte Carlo simulations to model uncertainty in our estimates.

Further details about our model and simulation approach including parameters, equations, assumptions, and additional sensitivity analyses are presented in Sections 1 and 4 in S1 Text.

### Outcome measures

Prior to vaccine availability–and also in scenarios where vaccines are available but immune-evasive variants are circulating–reducing viral transmission is an important public health policy objective. We calculated the number of averted infections and the percent reduction in the burden of infection to quantify the health impact of wearable sensor deployment. We defined the number of averted infections as the difference between the historical number of infections and the number of infections in a counterfactual scenario. We calculated the percent reduction in the burden of infection by dividing the number of averted infections by the historical number of infections. We also measured the number of days incorrectly spent in quarantine per month per device user (a consequence of false positive notifications) as the primary indicator of the strategy’s social burden [26]. Finally, to assess resource consumption, we quantified the number of additional lab-based tests (and rapid antigen tests, where applicable) required each day, on average.

## Results

### Baseline impact of wearable sensor deployment

**Fig 2:**
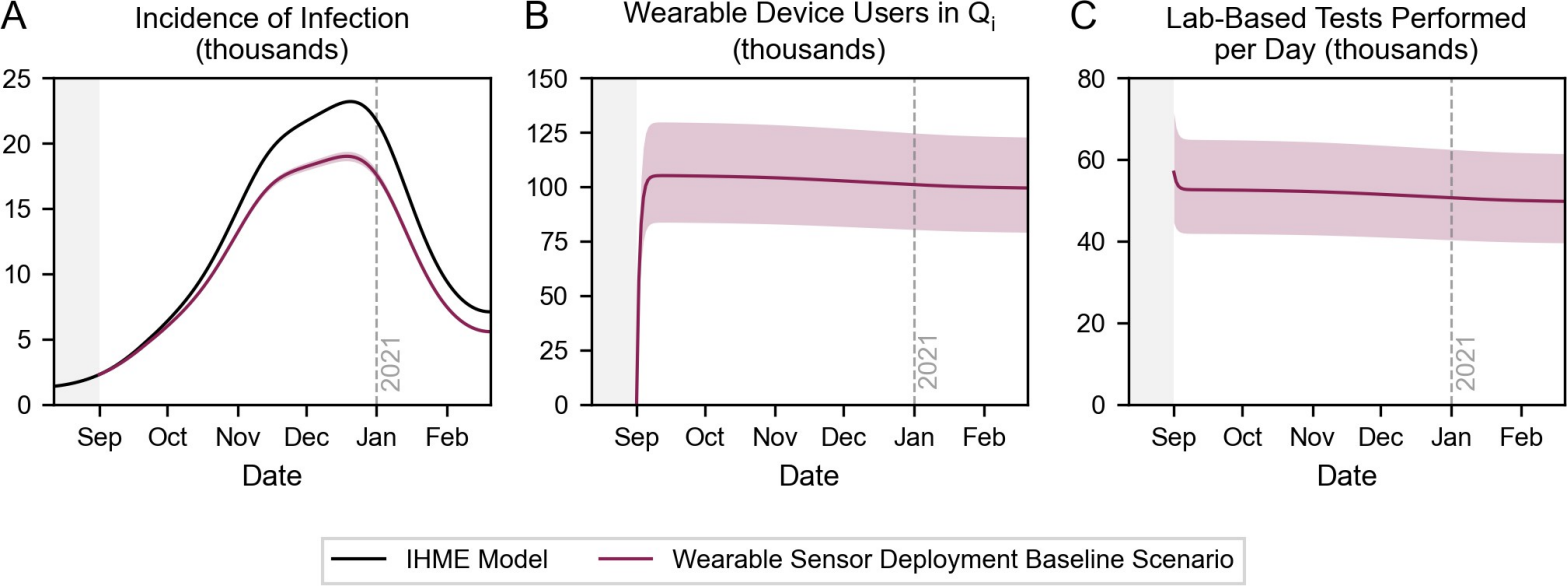
Baseline scenario for wearable sensor deployment. Time series depiction of (A) the incidence of infection, (B) the number of wearable device users incorrectly in quarantine, and (C) the daily demand for lab-based tests. Uptake, adherence, detection sensitivity, and detection specificity are set to 4%, 50%, 80%, and 92%, respectively.

We first investigated the baseline scenario in which detection algorithms that currently exist are made publicly available for device users to download and use (Fig 2) [10]. Upon notification of potential presymptomatic or asymptomatic infection, users are prompted to seek a confirmatory lab-based test, quarantine while awaiting the result (nominally, for 2 days), and self-isolate until recovery if positive. We used the nominal values outlined in Table B in S1 Text, setting uptake, adherence, detection sensitivity, and detection specificity to 4%, 50%, 80%, and 92%, respectively.

We observed that in a baseline scenario, 366,143 (95% CI: 333,242–396,944) infections could have been averted during Canada’s second COVID-19 wave–a 15.6% (95% CI: 14.2–16.9%) reduction in the burden of infection (Fig 2A). However, the social costs were high: between ~75,000 and ~125,000 device users were incorrectly quarantining on any given day (Fig 2B). Moreover, between ~40,000 and ~65,000 additional lab-based tests were required each day (Fig 2C), corresponding to a 51.6% (95% CI: 41.1–63.6%) increase in demand relative to historical volumes. Historically, ~101,000 lab-based tests were performed each day, on average, during the simulation timeframe [22,27]. The number of individuals incorrectly in quarantine and daily demand for lab-based tests were generally steady over time because they largely depend on the number of *Susceptible* device users, adherence, and detection specificity; the gradual decrease can be attributed to the flow of users into the *Removed* state. These findings were robust to our use of the IHME infection model (Fig B in S1 Text).

### Tradeoff between detection algorithm sensitivity and specificity

**Fig 3:**
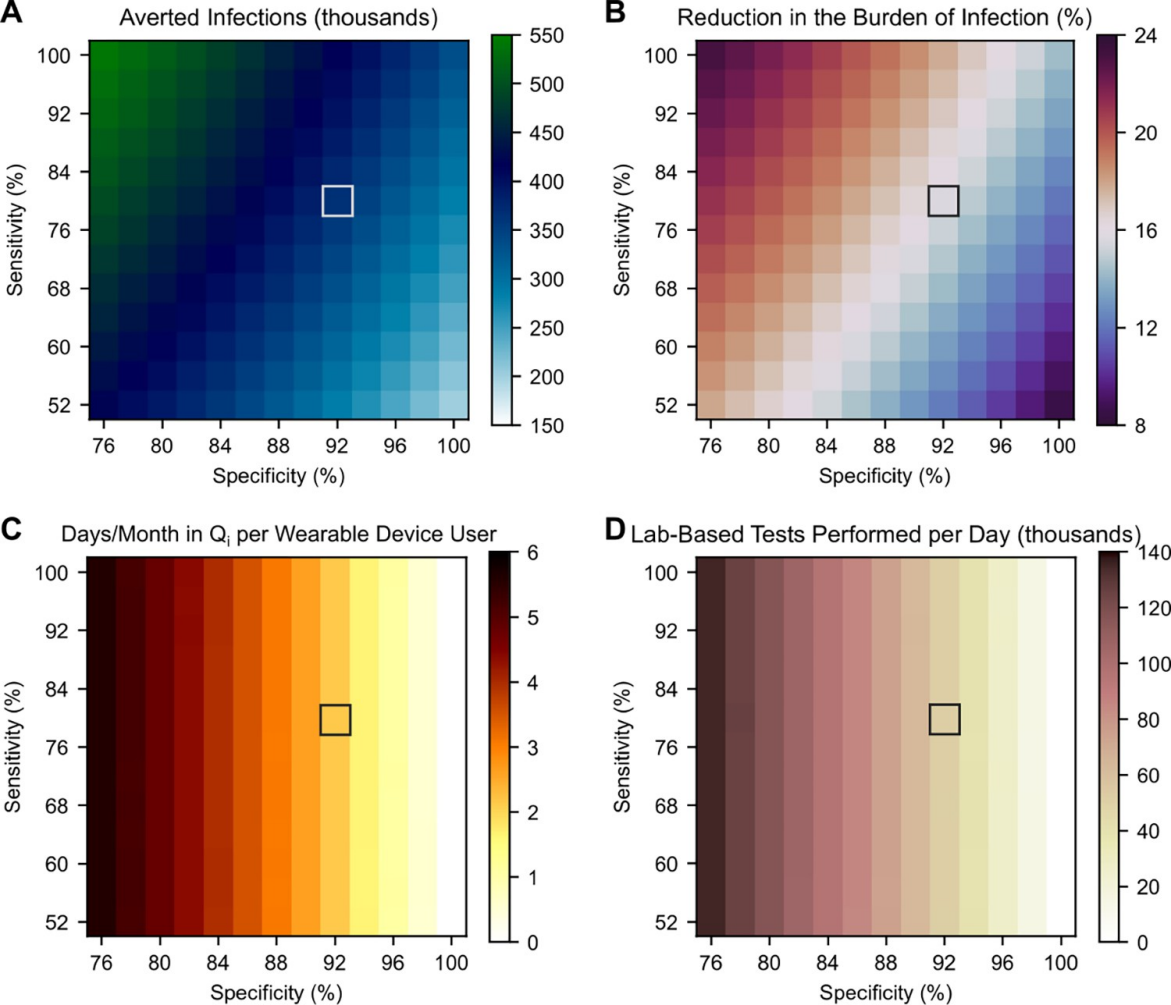
Tradeoff between detection sensitivity and specificity. (A) Averted infections, (B) reduction in the burden of infection, (C) days incorrectly spent in quarantine per month per user, and (D) average daily demand for lab-based tests, all over the simulation period, as a function of detection sensitivity and specificity. Grey boxes denote nominal sensitivity (80%) and specificity (92%).

After their initial release on technology platforms, health detection algorithms can be updated and improved as more real-world data are collected. However, it is often challenging to dramatically raise detection sensitivity and specificity at the same time. We explored the implications of this tradeoff (Fig 3), varying detection sensitivity and specificity while keeping uptake and adherence constant at 4% and 50%, respectively.

Increasing detection sensitivity increased the number of averted infections by prompting more *Infectious* users to quarantine (Fig 3A and Fig 3B). On the other hand, increasing specificity had a two-part effect. First, as specificity approached 100%, the number of days incorrectly spent in quarantine approached zero (Fig 3C); sensitivity had negligible impact on incorrect quarantines. Second, by virtue of decreasing the number of incorrect quarantines, increasing specificity resulted in a larger pool of *Susceptible* individuals; in turn, fewer infections were averted. Despite this second effect, incorrect quarantines were not central to the strategy’s public health impact. In the baseline scenario above (80% detection sensitivity, 4% uptake, and 50% adherence), a 12.1% (95% CI: 11.0–13.1%) reduction in the burden of infection was still achievable with perfect detection specificity (and no incorrect quarantines). 22.7% (95% CI: 13.1–32.5%) of averted infections could be attributed to incorrect quarantines in the baseline scenario, though this proportion decreased as sensitivity improved (Fig E in S1 Text).

In theory, increasing detection sensitivity would increase demand for lab-based tests. We found that this effect paled in comparison to the number of lab-based tests prompted by false positive notifications (Fig 3D). Lab-based test demand expectedly decreased as detection specificity increased.

### Impact of increasing uptake

**Fig 4:**
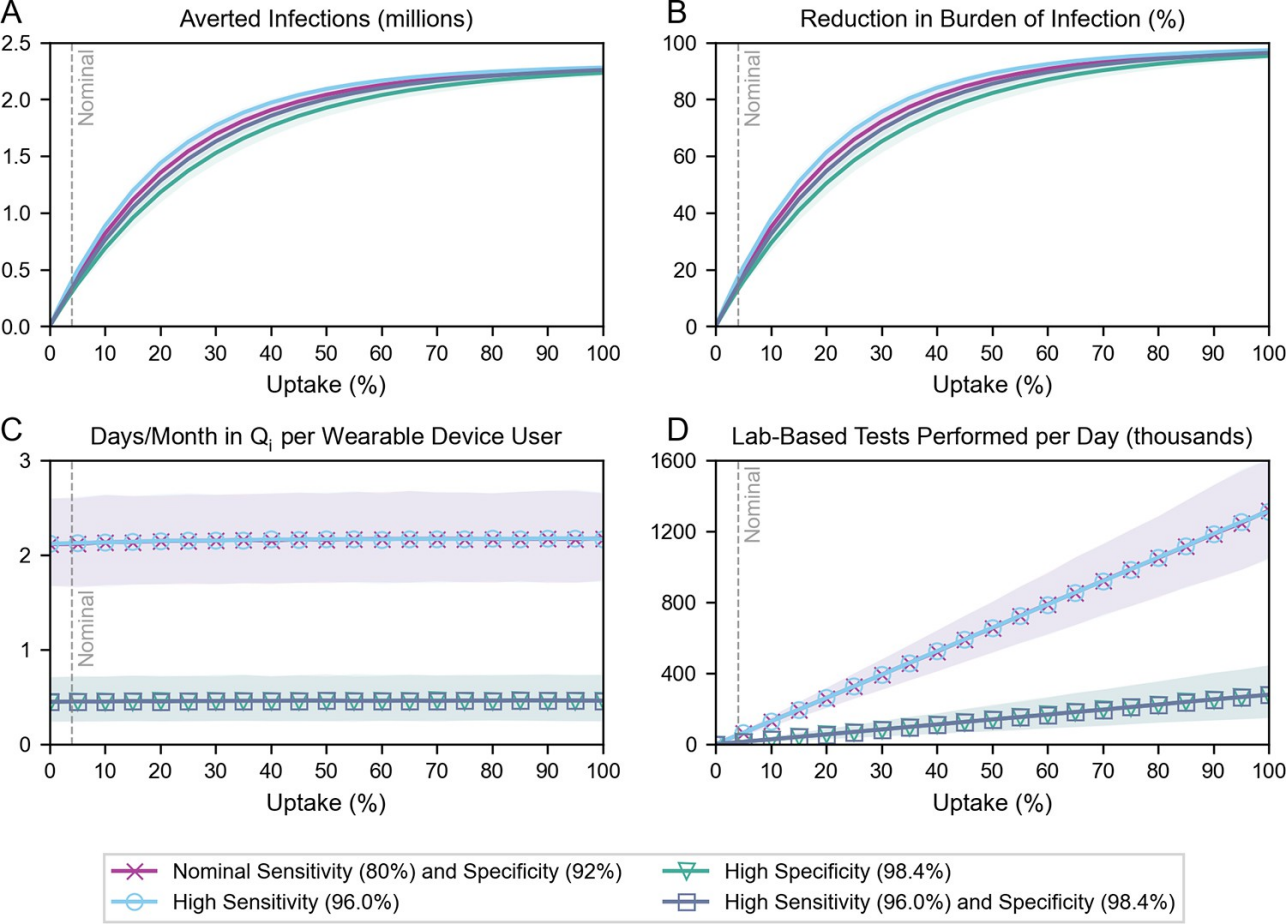
Impact of increasing uptake under different technology assumptions. (A) Averted infections, (B) reduction in the burden of infection, (C) days incorrectly spent in quarantine per month per user, and (D) average daily demand for lab-based tests, all over the simulation period, as a function of increasing uptake. Grey dashed lines denote nominal uptake (4%). In the “High Sensitivity” and “High Specificity” scenarios, detection specificity and sensitivity are kept at their nominal values, respectively. Symbol markers are added in (C) and (D) to distinguish overlapping curves: in these charts, the “Nominal Sensitivity and Specificity” and “High Sensitivity” curves overlap, and the “High Specificity” and “High Sensitivity and Specificity” curves overlap.

Ensuring that public health measures reach sufficient levels of uptake has been a continued challenge through the COVID-19 pandemic. Digital contact tracing and vaccination efforts have demonstrated that well-constructed policies–for example, incentivizing participation–can improve uptake of measures [28,29]. Here, we explored the role of uptake to provide relevant context for the design of wearable sensor deployment policies (Fig 4; Fig A in S1 Text). We estimated that uptake would fall between 0.5% and 7.5% (Tables B–D in S1 Text) at baseline but chose to present outcomes at all levels of uptake (i.e., from 0% to 100%) to illustrate emergent phenomena. We also explored multiple technology scenarios, setting “high” detection sensitivity and specificity at 96.0% and 98.4%, respectively; we based these increases on the respective goals of capturing 20% more infections and reducing the false positive rate by 80% relative to nominal values. We kept adherence constant at 50%.

In all technology scenarios, increasing uptake averted more infections, though with eventual diminishing returns (Fig 4A and Fig 4B). Within our estimated range of uptake (0.5% to 7.5%), and with nominal detection sensitivity and specificity, each percent increase in uptake resulted in an additional 3.4% (95% CI: 2.8–4.0%) reduction in the burden of infection (Fig 4B). As expected, improving detection specificity resulted in fewer averted infections when uptake was held constant; this effect was most pronounced between ~30% and ~60% uptake. The number of days incorrectly spent in quarantine per month per device user remained constant as a function of uptake but decreased from ~2.15 to ~0.45 when detection specificity was increased (Fig 4C). This ~80% decrease was consistent with our definition of “high specificity” underscoring that detection specificity directly influences the burden of incorrect quarantines on device users. The average daily demand for lab-based tests scaled linearly with uptake, but at a slower rate with improved detection specificity (Fig 4D).

### Impact of increasing adherence

**Fig 5:**
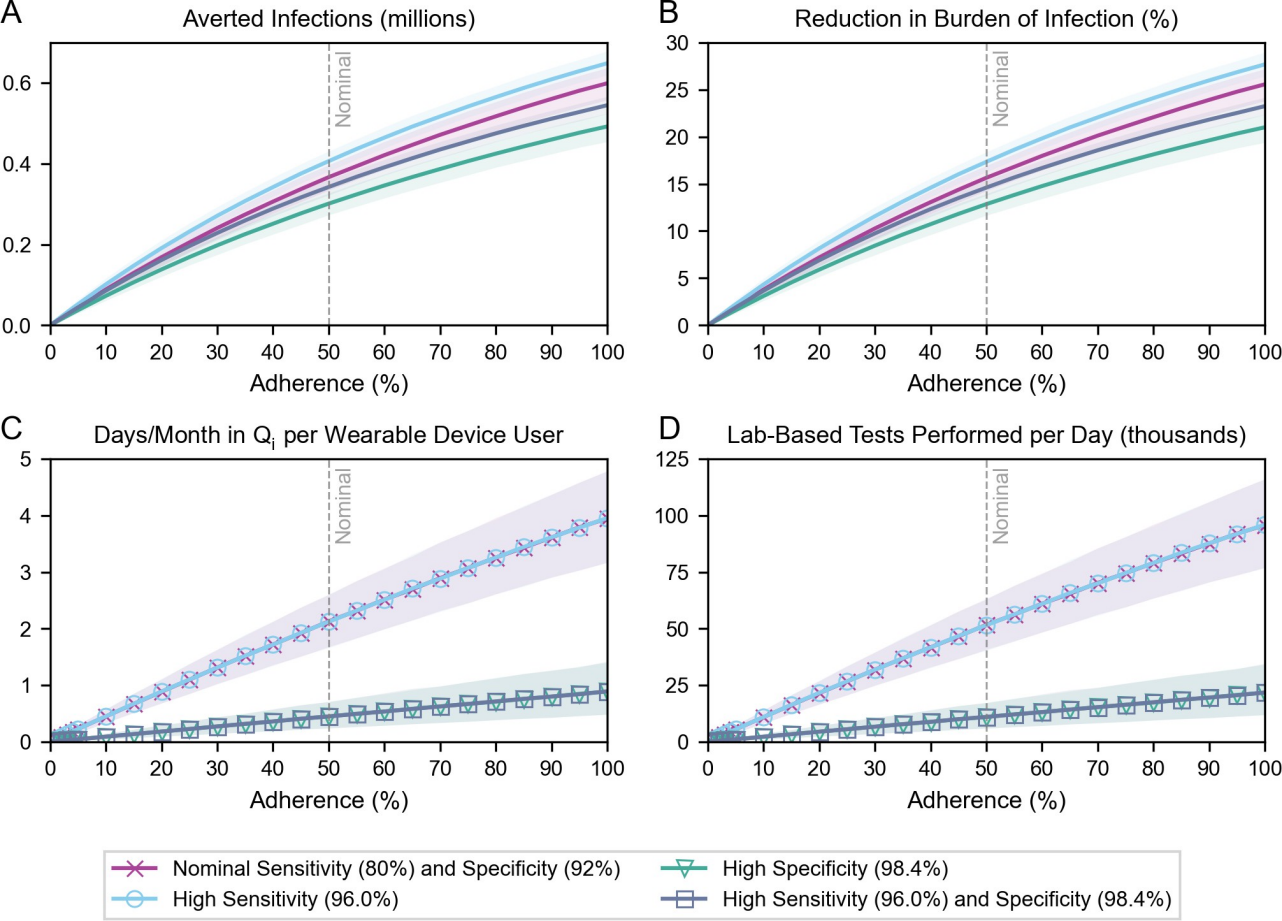
Impact of increasing adherence under different technology assumptions. (A) Averted infections, (B) reduction in the burden of infection, (C) days incorrectly spent in quarantine per month per user, and (D) average daily demand for lab-based tests, all over the simulation period, as a function of increasing adherence. Grey dashed lines denote nominal adherence (50%). In the “High Sensitivity” and “High Specificity” scenarios, detection specificity and sensitivity are kept at their nominal values, respectively. Symbol markers are added in (C) and (D) to distinguish overlapping curves: in these charts, the “Nominal Sensitivity and Specificity” and “High Sensitivity” curves overlap, and the “High Specificity” and “High Sensitivity and Specificity” curves overlap.

Adherence to public health guidelines also impacts the success of pandemic control measures. Targeted policies–for example, compensating individuals in self-isolation–could help improve compliance with public health recommendations [30]. Here, we explored the role of adherence in wearable sensor deployment strategies (Fig 5; Fig A in S1 Text). We captured adherence in one parameter to preserve model parsimony but recognize that there is likely to be great variation in the extent to which notified users adhere to recommended next steps in practice (Table B in S1 Text). For this reason, we chose to explore outcomes at all values of adherence–from 0% adherence, where no users comply with any recommended next steps, to 100% adherence, where all users comply with all recommended next steps. We kept uptake constant at 4% and assessed multiple technology scenarios using the same definitions of “high” detection sensitivity and specificity as before. Separately, we also considered the case of partial adherence where non-adherent users act more cautiously due to the notification (Fig C in S1 Text).

Adherence meaningfully impacted the achievable reduction in the burden of infection (Fig 5B). With nominal detection sensitivity and specificity, increasing adherence among participating wearable device users from 20% to 80% tripled the achieved reduction in the burden of infection, raising it from 7.2% (95% CI: 6.3–8.1%) to 22.1% (95% CI: 20.4–23.6%). However, increasing the proportion of users who comply with notifications also magnified the consequences of false positive notifications: the number of days incorrectly spent in quarantine per month per user (Fig 5C) and the demand for lab-based tests (Fig 5D) grew proportionally with adherence. These social and resource costs grew at a slower rate with improved detection specificity.

### Impact of offering confirmatory rapid antigen tests

**Fig 6:**
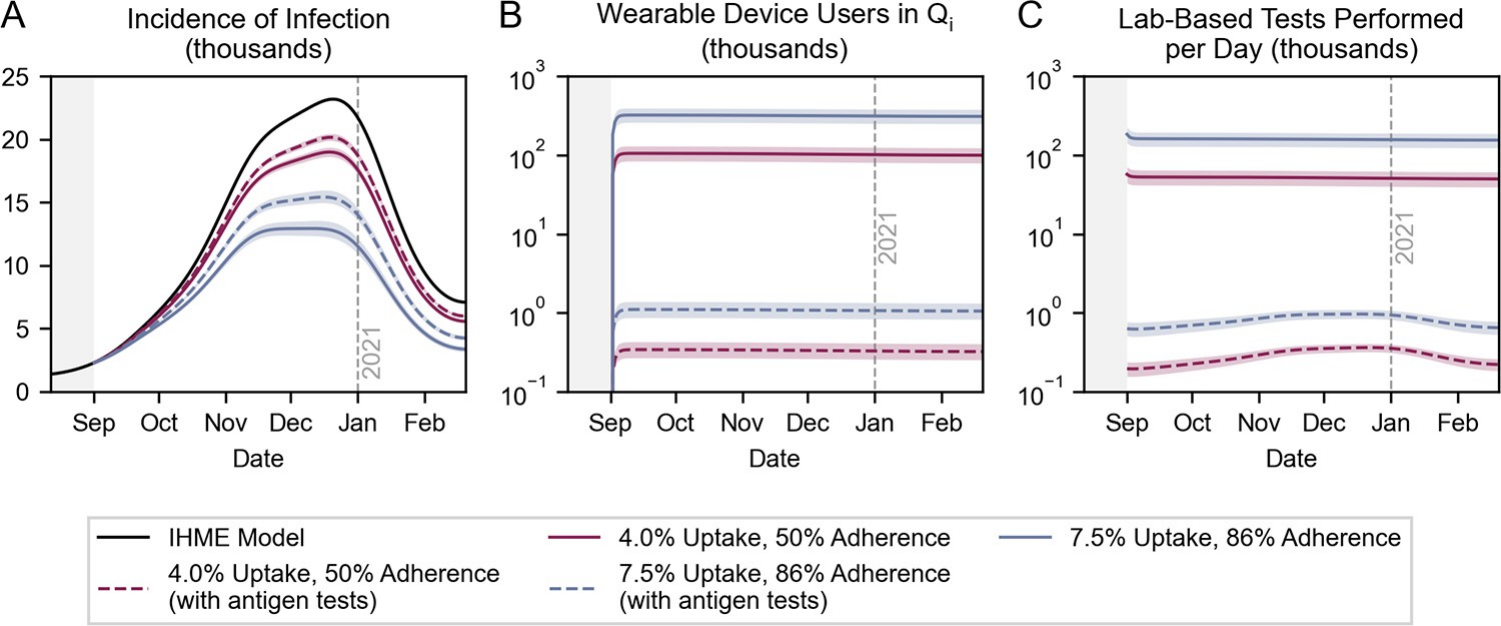
Wearable sensor deployment with confirmatory rapid antigen tests. Time series depiction of (A) the incidence of infection, (B) the number of wearable device users incorrectly in quarantine, and (C) the daily demand for lab-based tests. Detection sensitivity and specificity are set to their nominal values of 80% and 92%, respectively.

Our earlier findings suggested that false positive notifications of potential infection were the primary cause of unnecessary quarantines and lab-based tests. Improving detection specificity was one way to decrease false positive notifications. Here, we investigated whether offering confirmatory rapid antigen tests to users with a positive notification could also contribute to reducing unnecessary quarantines and lab-based tests (Fig 6; Table 1; Table E in S1 Text). We considered multiple scenarios, each with either low levels of uptake (0.5%) or adherence (14%), nominal levels of uptake (4%) or adherence (50%), or high levels of uptake (12.5%) or adherence (86%). We examined these scenarios in the cases of nominal detection sensitivity and specificity, and of “high” detection sensitivity and specificity (using the same definitions of “high” as above).

**Table 1:** Impact of offering confirmatory rapid antigen tests under different technology and behavioural assumptions. 95% confidence intervals are listed in parentheses. Table E in S1 Text depicts outcomes in analogous scenarios without rapid antigen tests.

Uptake (%)	Adherence (%)	Averted Infections (thousands)	Reduction in Burden of Infection (%)	Days/Month in Q_i_ per User (thousands)	Additional Lab-Based Tests Performed per Day	Additional Rapid Tests Performed per Day (thousands)
*Nominal Detection Sensitivity (80%) and Specificity (92%) Scenario*						
0.5	14	11.0 (9.4–12.6)	0.5 (0.4–0.5)	1.92 (1.47–2.46)	11 (9–13)	1.9 (1.5–2.5)
0.5	50	34.4 (30.9–37.6)	1.5 (1.3–1.6)	6.85 (5.29–8.55)	37 (32–43)	6.9 (5.4–8.7)
0.5	86	52.6 (47.9–56.5)	2.2 (2.0–2.4)	11.81 (9.23–14.68)	60 (52–69)	11.9 (9.3–14.8)
4.0	14	86.5 (73.5–99.5)	3.7 (3.1–4.2)	1.92 (1.46–2.45)	88 (73–103)	15.5 (11.8–19.8)
4.0	50	263.4 (237.1–286.6)	11.2 (10.1–12.2)	6.87 (5.34–8.53)	284 (244–326)	55.6 (43.2–69.0)
4.0	86	393.5 (362.2–420.3)	16.8 (15.5–17.9)	11.84 (9.29–14.79)	454 (391–527)	95.8 (75.2–119.6)
7.5	14	160.0 (136.9–183.9)	6.8 (5.8–7.8)	1.92 (1.46–2.46)	162 (135–191)	29.2 (22.2–37.3)
7.5	50	472.4 (427.8–511.5)	20.2 (18.3–21.8)	6.87 (5.32–8.60)	510 (438–592)	104.1 (80.7–130.3)
7.5	86	687.0 (636.8–728.4)	29.3 (27.2–31.1)	11.88 (9.24–14.79)	805 (686–936)	180.1 (140.2–224.2)
*High Detection Sensitivity (96*.*0%) and Specificity (98*.*4%) Scenario*						
0.5	14	13.0 (11.5–14.6)	0.6 (0.5–0.6)	0.38 (0.21–0.63)	7 (6–9)	0.4 (0.2–0.6)
0.5	50	39.9 (37.6–41.9)	1.7 (1.6–1.8)	1.37 (0.73–2.18)	23 (21–26)	1.4 (0.8–2.2)
0.5	86	59.9 (57.6–61.7)	2.6 (2.5–2.6)	2.35 (1.26–3.74)	35 (31–39)	2.4 (1.3–3.8)
4.0	14	102.6 (91.1–114.6)	4.4 (3.9–4.9)	0.39 (0.21–0.63)	58 (50–67)	3.2 (1.7–5.2)
4.0	50	303.0 (286.6–317.5)	12.9 (12.2–13.5)	1.37 (0.72–2.20)	167 (149–188)	11.2 (5.9–17.9)
4.0	86	443.2 (426.2–455.3)	18.9 (18.2–19.4)	2.35 (1.26–3.73)	241 (215–276)	19.2 (10.4–30.4)
7.5	14	188.8 (167.3–210.9)	8.1 (7.1–9.0)	0.38 (0.20–0.62)	105 (91–121)	5.9 (3.2–9.5)
7.5	50	539.4 (511.4–562.9)	23.0 (21.8–24.0)	1.38 (0.74–2.23)	284 (253–324)	21.1 (11.5–34.0)
7.5	86	767.3 (741.7–785.9)	32.7 (31.6–33.5)	2.37 (1.24–3.82)	395 (343–461)	36.2 (19.1–58.1)

The use of antigen tests reduced the number of days incorrectly spent in quarantine by ~300-fold by increasing the “effective specificity” of the strategy (Fig 6B). That is, with antigen tests, the likelihood of a *Susceptible* user being incorrectly prompted to quarantine on a given day fell from (1 –ν_w_) to the product of (1 –ν_w_) and (1 –ν_a_), where ν_w_ and ν_a_ are detection algorithm specificity and antigen test specificity, respectively. In earlier scenarios (Fig 4A and 5A), the number of averted infections was decreased by improving detection specificity more than it was increased by improving detection sensitivity; fewer infections were averted in scenarios with “high” as opposed to nominal detection sensitivity and specificity. Here, the specificity contributed by the antigen tests diminished the relative impact of improving detection specificity on the number of averted infections: the “effective specificity” of the strategy was 99.976% with nominal detection specificity and 99.995% with high detection specificity (Table B in S1 Text) [31]. Instead, improving detection sensitivity was what increased the number of averted infections. Importantly, antigen tests had the secondary effect of decreasing the strategy’s “effective sensitivity”–the product of antigen test sensitivity (91.1%) and detection algorithm sensitivity [31].

Offering confirmatory rapid antigen tests also decreased the demand for lab-based tests by ~200-fold, alleviating the burden on testing infrastructure (Fig 6C). We earlier found that in a baseline scenario (4% uptake, 50% adherence, 80% detection sensitivity, 92% detection specificity), between ~40,000 and ~65,000 additional lab-based tests would be required each day (Fig 2C). Here, in an analogous scenario, only 284 (95% CI: 244–326) additional lab-based tests would be required each day, on average, and 55,600 (95% CI: 43,200–69,000) antigen tests would be performed instead (Table 1).

## Discussion

We used a counterfactual model of Canada’s second COVID-19 wave to demonstrate that wearable sensors capable of detecting infections before or absent symptoms have meaningful potential to help mitigate the acute phase of a pandemic. Through continuous and non-invasive monitoring of physiological parameters, these devices can help FTTI systems identify hidden infection chains with minimal delay and without active user engagement or broad sharing of user data. We showed that (1) deploying currently available detection algorithms could have helped reduce the acute phase burden of infection, but with substantial social and resource costs; (2) improving detection algorithm specificity and offering confirmatory rapid antigen tests can help minimize unnecessary quarantines and lab-based tests; and (3) once false positive notifications are minimized, increasing uptake and adherence become effective strategies to scale the number of averted infections.

In theory, wearable sensor deployment reduces the burden of infection by decreasing the pool of *Infectious* individuals (a function of detection algorithm sensitivity). Here we found that detection specificity played an unexpectedly large role as well, with false positive notifications of potential infection prompting unnecessary quarantines and thereby decreasing the pool of *Susceptible* individuals. Thus, although prioritizing uptake and adherence as part of a wearable sensor deployment strategy could mitigate a substantial number of infections, the unsustainable growth of associated costs should also be considered. In a baseline scenario, without improvements to detection specificity, every user would spend over two days a month on average incorrectly quarantining, and ~40,000 to ~65,000 additional confirmatory lab-based tests would be required each day. The social and economic harm caused by solely promoting uptake or adherence without improvements to detection specificity would likely undermine public confidence in and compliance with a wearable-based pandemic mitigation strategy [32]. Alavi *et al* found that many false positives were due to the detection algorithm identifying lifestyle-driven changes in resting heart rate (e.g., after intense exercise or alcohol consumption); accounting for these factors using more advanced algorithms may be one way to target improved detection specificity [10].

We found that the inclusion of confirmatory antigen testing was a valuable mechanism, beyond improving detection specificity, to increase the “effective specificity” of the strategy and decrease the overall false positive rate. The inclusion of antigen testing decreased days incorrectly spent in quarantine by ~300-fold and brought the additional demand on lab-based testing infrastructure to justifiable levels. However, even with the inclusion of antigen tests, improvements to detection specificity still had value. In scenarios with “high” nominal detection specificity, we observed a ~4-fold reduction in days incorrectly spent in quarantine per month per user, a ~2-fold reduction in lab-based tests performed each day, and a ~5-fold reduction in antigen tests used each day. Importantly, a strategy in which antigen tests support the deployment of wearable sensors is notably different from one involving frequent use of rapid antigen tests for diagnosis or screening [33]. On their own, broad antigen test-based screening approaches require tremendous manufacturing volumes, infrastructure, and funding [34]. Conversely, wearable sensors can non-invasively detect infections without active user engagement, reducing the effort required to participate. Further, wearable sensors may even help improve diagnostic test allocation by directing tests toward individuals with a higher pre-test probability of infection [35].

The COVID-19 pandemic’s evolution has been shaped by the uptake of vaccines, the emergence of more transmissible and immune-evasive variants, and the potential for breakthrough and repeat infections [36]. Although we did not consider these factors when modelling Canada’s second wave, we speculate that their effects on wearable sensor-based mitigation strategies would be driven by changes in users’ physiological responses and in SARS-CoV-2 epidemiology. In particular, we hypothesize that wearable sensor-based mitigation would be impacted in four major ways. First, vaccination has been found to elicit similar physiological responses to infection (e.g., elevated resting heart rate) and these physiological responses might be captured by wearable sensor-based detection algorithms [10,37]. We expect this to manifest as an increase in the incidence of false positive notifications, which we have considered in depth in our analyses related to detection specificity. However, we also speculate that vaccination-driven false positive notifications would likely be flagged as such by the user and ignored. Second, prior immunity from vaccination may attenuate physiological responses elicited by breakthrough infections, altering detection sensitivity [38]. Although it might generally be expected that the degree of attenuation would depend on the VOC causing infection, as well as the specific infection and vaccination history of the individual, evidence of minimal differences between physiological responses to breakthrough infections during Germany’s Delta and Omicron waves has been reported [38]. From a modeling perspective, incorporating temporal changes in detection sensitivity may be an appropriate starting point for exploring this effect. Third, the onset of symptoms may occur earlier in the infectious period in individuals with pre-existing immunity than in immunologically naïve individuals [39,40]. In these scenarios, the early onset of symptoms would already contribute to the detection of infections earlier in the infectious period. However, we speculate that if detection algorithms retained their ability to identify presymptomatic infections, wearable sensors could even further reduce the fraction of the infectious period in which users unknowingly transmit the virus–and in turn, even further decrease the burden of infection. Finally, increases in transmissibility–whether due to higher viral loads or immune evasion in VOCs–would also influence the impact of wearable sensor-based mitigation strategies by attenuating the achievable reduction in the burden of infection (Fig H in S1 Text) [3,41–43]. Moving forward, more empirical data will be needed in order to develop models of wearable sensor deployment in the SARS-CoV-2 vaccine and variant era, and in turn explore these hypotheses.

Our work has important limitations. First, we do not account for heterogeneities in wearable device use which, in reality, is influenced by age, race, level of education, and income [44,45]. Future analyses could more precisely address how a device user being removed from the pool of *Susceptible* or *Infectious* individuals will impact the epidemic trajectory based on that user’s demographic and socioeconomic profile. Indeed, the COVID-19 pandemic has disproportionately impacted low-income and minority groups, while younger individuals are more likely to be super-spreaders [46–48]. Future studies could also consider policies that subsidize wearable devices, reducing the participation barrier for groups underrepresented among current device owners. Second, we made the simplifying assumption that all users without symptoms (and that no users with symptoms) could benefit from wearable-informed prompts to seek a confirmatory test and tentatively quarantine. We may be underestimating the effect size because wearable sensors also show promise in detecting symptomatic SARS-CoV-2 infection and many symptomatic individuals did not historically self-isolate [16–18,49,50]. Third, we did not consider how uptake or adherence may vary with time, detection accuracy, or other factors [28,32,49,51]. Finally, we did not consider how detection algorithm performance varies over the course of infection.

Using the example of COVID-19, we demonstrated the potential of wearable sensors to support FTTI systems with real-time detection of presymptomatic and asymptomatic infections and thereby reduce the burden of infection during a pandemic. Messaging to the public will be an important to ensure a wearable sensor-based mitigation strategy is successful: for example, public health leaders will need to communicate the limitations of wearable sensors with respect to detecting infections and emphasize that a lack of a notification does not rule out potential infection (Fig C in S1 Text). Moreover, moving forward, it will also be important to consider how wearable sensor data can be linked with other health data such as laboratory tests to yield more impactful diagnoses, to address potential issues with data format and secure storage with an eye to heightened challenges in resource-constrained settings, and to ensure that device users prompted to quarantine have appropriate supports to do so [30,52,53]. Ultimately, as sensor technology and detection algorithms evolve–for example, to potentially distinguish infections with SARS-CoV-2 from those with seasonal influenza–there is clear merit to further exploring how wearable sensors can be incorporated into FTTI systems to support pandemic mitigation [54].

## Supporting information

S1 Text The file “S1_Text.pdf” contains additional information about study methodology, supplementary results, and sensitivity analyses. (PDF)

## References

[1] Rajan S, D.Cylus J, Mckee M. What do countries need to do to implement effective ‘find, test, trace, isolate and support’ systems? J R Soc Med. 2020;113: 245–250. doi: 10.1177/0141076820939395 32663428 PMC7361659

[2] Contreras S, Dehning J, Loidolt M, Zierenberg J, Spitzner FP, Urrea-Quintero JH, et al. The challenges of containing SARS-CoV-2 via test-trace-and-isolate. Nat Commun. 2021;12: 378. doi: 10.1038/s41467-020-20699-8 33452267 PMC7810722

[3] Grantz KH, Lee EC, D’Agostino McGowan L, Lee KH, Metcalf CJE, Gurley ES, et al. Maximizing and evaluating the impact of test-trace-isolate programs: A modeling study. PLOS Med. 2021;18: e1003585. doi: 10.1371/journal.pmed.1003585 33930019 PMC8121292

[4] Lewis D. Why many countries failed at COVID contact-tracing—but some got it right. Nature. 14 Nov 2020. Available: https://www.nature.com/articles/d41586-020-03518-4. Accessed 17 Dec 2021. doi: 10.1038/d41586-020-03518-4 33318682

[5] Rubin R. The Challenges of Expanding Rapid Tests to Curb COVID-19. JAMA. 2020;324: 1813. doi: 10.1001/jama.2020.21106 33084882

[6] Freeze C. Supply crunch coming as demand for COVID-19 rapid testing soars. The Globe and Mail. 31 Dec 2021. Available: https://www.theglobeandmail.com/canada/article-supply-crunch-coming-as-demand-for-covid-19-rapid-testing-soars/. Accessed 1 Jan 2022.

[7] Li X, Dunn J, Salins D, Zhou G, Zhou W, Schüssler-Fiorenza Rose SM, et al. Digital Health: Physiomes Tracking and Activity Using Wearable Biosensors Reveals Useful Health-Related Information. Kirkwood T, editor. PLOS Biol. 2017;15: e2001402. doi: 10.1371/journal.pbio.2001402 28081144 PMC5230763

[8] Grzesiak E, Bent B, McClain MT, Woods CW, Tsalik EL, Nicholson BP, et al. Assessment of the Feasibility of Using Noninvasive Wearable Biometric Monitoring Sensors to Detect Influenza and the Common Cold Before Symptom Onset. JAMA Netw Open. 2021;4: e2128534. doi: 10.1001/jamanetworkopen.2021.28534 34586364 PMC8482058

[9] Mishra T, Wang M, Metwally AA, Bogu GK, Brooks AW, Bahmani A, et al. Pre-symptomatic detection of COVID-19 from smartwatch data. Nat Biomed Eng. 2020;4: 1208–1220. doi: 10.1038/s41551-020-00640-6 33208926 PMC9020268

[10] Alavi A, Bogu GK, Wang M, Rangan ES, Brooks AW, Wang Q, et al. Real-time alerting system for COVID-19 and other stress events using wearable data. Nat Med. 2021 [cited 15 Dec 2021]. doi: 10.1038/s41591-021-01593-2 34845389 PMC8799466

[11] Radin JM, Wineinger NE, Topol EJ, Steinhubl SR. Harnessing wearable device data to improve state-level real-time surveillance of influenza-like illness in the USA: a population-based study. Lancet Digit Health. 2020;2: e85–e93. doi: 10.1016/S2589-7500(19)30222-5 33334565 PMC8048388

[12] Zhu G, Li J, Meng Z, Yu Y, Li Y, Tang X, et al. Learning from Large-Scale Wearable Device Data for Predicting the Epidemic Trend of COVID-19. Ren J, editor. Discrete Dyn Nat Soc. 2020;2020: 1–8. doi: 10.1155/2020/6152041

[13] Layton AT, Sadria M. Understanding the dynamics of SARS-CoV-2 variants of concern in Ontario, Canada: a modeling study. Sci Rep. 2022;12: 2114. doi: 10.1038/s41598-022-06159-x 35136161 PMC8826311

[14] Lumley SF, O’Donnell D, Stoesser NE, Matthews PC, Howarth A, Hatch SB, et al. Antibody Status and Incidence of SARS-CoV-2 Infection in Health Care Workers. N Engl J Med. 2021;384: 533–540. doi: 10.1056/NEJMoa2034545 33369366 PMC7781098

[15] Government of Canada. Testing for COVID-19: How and where we test for active infections. 2021 Dec. Available: https://www.canada.ca/en/public-health/services/diseases/2019-novel-coronavirus-infection/symptoms/testing/diagnosing.html.

[16] Quer G, Radin JM, Gadaleta M, Baca-Motes K, Ariniello L, Ramos E, et al. Wearable sensor data and self-reported symptoms for COVID-19 detection. Nat Med. 2021;27: 73–77. doi: 10.1038/s41591-020-1123-x 33122860

[17] Gadaleta M, Radin JM, Baca-Motes K, Ramos E, Kheterpal V, Topol EJ, et al. Passive detection of COVID-19 with wearable sensors and explainable machine learning algorithms. Npj Digit Med. 2021;4: 166. doi: 10.1038/s41746-021-00533-1 34880366 PMC8655005

[18] Hirten RP, Danieletto M, Tomalin L, Choi KH, Zweig M, Golden E, et al. Use of Physiological Data From a Wearable Device to Identify SARS-CoV-2 Infection and Symptoms and Predict COVID-19 Diagnosis: Observational Study. J Med Internet Res. 2021;23: e26107. doi: 10.2196/26107 33529156 PMC7901594

[19] Poongodi M, Hamdi M, Malviya M, Sharma A, Dhiman G, Vimal S. Diagnosis and combating COVID-19 using wearable Oura smart ring with deep learning methods. Pers Ubiquitous Comput. 2022;26: 25–35. doi: 10.1007/s00779-021-01541-4 33654480 PMC7908947

[20] Bobrovitz N, Arora RK, Cao C, Boucher E, Liu M, Donnici C, et al. Global seroprevalence of SARS-CoV-2 antibodies: A systematic review and meta-analysis. Khudyakov YE, editor. PLOS ONE. 2021;16: e0252617. doi: 10.1371/journal.pone.0252617 34161316 PMC8221784

[21] Wu SL, Mertens AN, Crider YS, Nguyen A, Pokpongkiat NN, Djajadi S, et al. Substantial underestimation of SARS-CoV-2 infection in the United States. Nat Commun. 2020;11: 4507. doi: 10.1038/s41467-020-18272-4 32908126 PMC7481226

[22] Institute for Health Metrics and Evaluation (IHME). COVID-19 Projections. Seattle, WA: IHME, University of Washington; 2020. Available: https://covid19.healthdata.org/projections.

[23] Barber RM, Sorensen RJD, Pigott DM, Bisignano C, Carter A, Amlag JO, et al. Estimating global, regional, and national daily and cumulative infections with SARS-CoV-2 through Nov 14, 2021: a statistical analysis. The Lancet. 2022; S0140673622004846. doi: 10.1016/S0140-6736(22)00484-6 35405084 PMC8993157

[24] Walker PGT, Whittaker C, Watson OJ, Baguelin M, Winskill P, Hamlet A, et al. The impact of COVID-19 and strategies for mitigation and suppression in low- and middle-income countries. Science. 2020;369: 413–422. doi: 10.1126/science.abc0035 32532802 PMC7292504

[25] Cleary JL, Fang Y, Sen S, Wu Z. A Caveat to Using Wearable Sensor Data for COVID-19 Detection: The Role of Behavioral Change after Receipt of Test Results. Infectious Diseases (except HIV/AIDS); 2021 Apr. doi: 10.1101/2021.04.17.21255513 36584148 PMC9803125

[26] Fearon E, Buchan IE, Das R, Davis EL, Fyles M, Hall I, et al. SARS-CoV-2 antigen testing: weighing the false positives against the costs of failing to control transmission. Lancet Respir Med. 2021;9: 685–687. doi: 10.1016/S2213-2600(21)00234-4 34139150 PMC8203180

[27] Government of Canada. COVID-19 daily epidemiology update. Available: https://health-infobase.canada.ca/covid-19/epidemiological-summary-covid-19-cases.html.

[28] Munzert S, Selb P, Gohdes A, Stoetzer LF, Lowe W. Tracking and promoting the usage of a COVID-19 contact tracing app. Nat Hum Behav. 2021;5: 247–255. doi: 10.1038/s41562-020-01044-x 33479505

[29] Vavreck L. $100 as Incentive to Get a Shot? Experiment Suggests It Can Pay Off. The New York Times. 4 May 2021. Available: https://www.nytimes.com/2021/05/04/upshot/vaccine-incentive-experiment.html. Accessed 17 Dec 2021.

[30] Bodas M, Peleg K. Income assurances are a crucial factor in determining public compliance with self-isolation regulations during the COVID-19 outbreak–cohort study in Israel. Isr J Health Policy Res. 2020;9: 54. doi: 10.1186/s13584-020-00418-w 33081833 PMC7573868

[31] Abbot. In vitro diagnostic rapid test for qualitative detection of SARS-CoV-2 antigen (Ag). Abbot; Available: https://www.globalpointofcare.abbott/en/product-details/panbio-covid-19-ag-antigen-test.html.

[32] Kaptchuk G, Goldstein DG, Hargittai E, Hofman J, Redmiles EM. How good is good enough for COVID19 apps? The influence of benefits, accuracy, and privacy on willingness to adopt. ArXiv200504343 Cs. 2020 [cited 19 Dec 2021]. Available: http://arxiv.org/abs/2005.04343.

[33] Larremore DB, Wilder B, Lester E, Shehata S, Burke JM, Hay JA, et al. Test sensitivity is secondary to frequency and turnaround time for COVID-19 screening. Sci Adv. 2021;7: eabd5393. doi: 10.1126/sciadv.abd5393 33219112 PMC7775777

[34] Iacobucci G. Covid-19: Government rolls out twice weekly rapid testing to all in England. BMJ. 2021; n902. doi: 10.1136/bmj.n902 33824178

[35] Dunn J, Shandhi MMH, Cho P, Roghanizad A, Singh K, Wang W, et al. A Method for Intelligent Allocation of Diagnostic Testing by Leveraging Data from Commercial Wearable Devices: A Case Study on COVID-19. In Review; 2022 Apr. doi: 10.21203/rs.3.rs-1490524/v1 36050372 PMC9434073

[36] Marcelin JR, Pettifor A, Janes H, Brown ER, Kublin JG, Stephenson KE. COVID-19 Vaccines and SARS-CoV-2 Transmission in the Era of New Variants: A Review and Perspective. Open Forum Infect Dis. 2022;9: ofac124. doi: 10.1093/ofid/ofac124 35493113 PMC8992234

[37] Quer G, Gadaleta M, Radin JM, Andersen KG, Baca-Motes K, Ramos E, et al. Inter-individual variation in objective measure of reactogenicity following COVID-19 vaccination via smartwatches and fitness bands. Npj Digit Med. 2022;5: 49. doi: 10.1038/s41746-022-00591-z 35440684 PMC9019018

[38] Wiedermann M, Rose AH, Maier BF, Kolb JJ, Hinrichs D, Brockmann D. Evidence for positive long- and short-term effects of vaccinations against COVID-19 in wearable sensor metrics—Insights from the German Corona Data Donation Project.: 17. 10.1093/pnasnexus/pgad223 PMC10368316 37497048

[39] Landon E, Bartlett AH, Marrs R, Guenette C, Weber SG, Mina MJ. High Rates of Rapid Antigen Test Positivity After 5 days of Isolation for COVID-19. Infectious Diseases (except HIV/AIDS); 2022 Feb. doi: 10.1101/2022.02.01.22269931

[40] Lefferts B, Blake I, Bruden D, Hagen MB, Hodges E, Kirking HL, et al. Antigen Test Positivity After COVID-19 Isolation—Yukon-Kuskokwim Delta Region, Alaska, January–February 2022. MMWR Morb Mortal Wkly Rep. 2022;71: 293–298. doi: 10.15585/mmwr.mm7108a3 35202352

[41] Puhach O, Adea K, Hulo N, Sattonnet P, Genecand C, Iten A, et al. Infectious viral load in unvaccinated and vaccinated individuals infected with ancestral, Delta or Omicron SARS-CoV-2. Nat Med. 2022 [cited 12 Jul 2022]. doi: 10.1038/s41591-022-01816-0 35395151

[42] Kozlov M. How does Omicron spread so fast? A high viral load isn’t the answer. Nature. 2022; d41586-022-00129-z. doi: 10.1038/d41586-022-00129-z 35046586

[43] Chin ET, Huynh BQ, Chapman LAC, Murrill M, Basu S, Lo NC. Frequency of Routine Testing for Coronavirus Disease 2019 (COVID-19) in High-risk Healthcare Environments to Reduce Outbreaks. Clin Infect Dis. 2021;73: e3127–e3129. doi: 10.1093/cid/ciaa1383 33570097 PMC7797732

[44] Chandrasekaran R, Katthula V, Moustakas E. Patterns of Use and Key Predictors for the Use of Wearable Health Care Devices by US Adults: Insights from a National Survey. J Med Internet Res. 2020;22: e22443. doi: 10.2196/22443 33064083 PMC7600024

[45] Vogels E. About one-in-five Americans use a smart watch or fitness tracker. Pew Research Centre. Available: https://www.pewresearch.org/fact-tank/2020/01/09/about-one-in-five-americans-use-a-smart-watch-or-fitness-tracker/.

[46] Liao TF, De Maio F. Association of Social and Economic Inequality With Coronavirus Disease 2019 Incidence and Mortality Across US Counties. JAMA Netw Open. 2021;4: e2034578. doi: 10.1001/jamanetworkopen.2020.34578 33471120 PMC7818127

[47] Lau MSY, Grenfell B, Thomas M, Bryan M, Nelson K, Lopman B. Characterizing superspreading events and age-specific infectiousness of SARS-CoV-2 transmission in Georgia, USA. Proc Natl Acad Sci. 2020;117: 22430–22435. doi: 10.1073/pnas.2011802117 32820074 PMC7486752

[48] Lemieux JE, Siddle KJ, Shaw BM, Loreth C, Schaffner SF, Gladden-Young A, et al. Phylogenetic analysis of SARS-CoV-2 in Boston highlights the impact of superspreading events. Science. 2021;371: eabe3261. doi: 10.1126/science.abe3261 33303686 PMC7857412

[49] Smith LE, Potts HWW, Amlôt R, Fear NT, Michie S, Rubin GJ. Adherence to the test, trace, and isolate system in the UK: results from 37 nationally representative surveys. BMJ. 2021; n608. doi: 10.1136/bmj.n608 33789843 PMC8010268

[50] Carlsen EØ, Caspersen IH, Trogstad L, Gjessing HK, Magnus P. Public adherence to governmental recommendations regarding quarantine and testing for COVID-19 in two Norwegian cohorts. Epidemiology; 2020 Dec. doi: 10.1101/2020.12.18.20248405

[51] Wright L, Steptoe A, Fancourt D. Predictors of self-reported adherence to COVID-19 guidelines. A longitudinal observational study of 51,600 UK adults. Lancet Reg Health—Eur. 2021;4: 100061. doi: 10.1016/j.lanepe.2021.100061 33997831 PMC7907734

[52] Chung S-C, Marlow S, Tobias N, Alogna A, Alogna I, You S-L, et al. Lessons from countries implementing find, test, trace, isolation and support policies in the rapid response of the COVID-19 pandemic: a systematic review. BMJ Open. 2021;11: e047832. doi: 10.1136/bmjopen-2020-047832 34187854 PMC8251680

[53] Xu S, Rwei AY, Vwalika B, Chisembele MP, Stringer JSA, Ginsburg AS, et al. Wireless skin sensors for physiological monitoring of infants in low-income and middle-income countries. Lancet Digit Health. 2021;3: e266–e273. doi: 10.1016/S2589-7500(21)00001-7 33640306

[54] Yanamala N, Krishna NH, Hathaway QA, Radhakrishnan A, Sunkara S, Patel H, et al. A vital sign-based prediction algorithm for differentiating COVID-19 versus seasonal influenza in hospitalized patients. Npj Digit Med. 2021;4: 95. doi: 10.1038/s41746-021-00467-8 34088961 PMC8178379

