# Supplementary material for: Deploying wearable sensors for pandemic mitigation: A counterfactual modelling study of Canada’s second COVID-19 wave
Source: PLOS Digit Health. 2022 Sep 6;1(9):e0000100. doi: 10.1371/journal.pdig.0000100 (PMC9931244; doi:10.1371/journal.pdig.0000100)
Supplement: S1 Text — (PDF) [file pdig.0000100.s001.pdf]

Supporting Information (SI): S1 Text

1. Modeling assumptions, variables, and equations

Table A: SARS-CoV-2 characteristics. We assumed a mean generation time of 7 days [1–3]. We assumed a mean incubation period of 5 days [1–4]. We applied the fact that mean generation time is equal to the sum of the mean latent period and the mean infectious period [5]. We subtracted the mean latent period from the mean incubation period to obtain the presymptomatic infectious period. We subtracted the incubation period from the mean generation time to obtain the mean asymptomatic and symptomatic infectious period. During Monte Carlo simulations, we modelled asymptomatic prevalence as a beta random variable with a mean of 0.4 and a sample size of 200; a sample size of 200 is greater than 75% of the study populations examined by Oran and colleagues [6].

**Parameter**	**Symbol**	**Value**
Latent period	$1 / \alpha$	3 days [1,7,8]
Presymptomatic infectious period	$1 / \tau$	2 days [1–4,7,8]
Asymptomatic and symptomatic infectious period	$1 / \gamma$	2 days [1–4]
Asymptomatic prevalence	ρ	40% [6]
Transmission potential without symptoms relative to with symptoms	λ	50% [9–11]
Daily new infections	π	varies
Average number of transmissions per infectious person per day	β	varies

Table B: Wearable device and policy parameters and their associated assumptions.

**Parameter**	**Symbol**	**Nominal Value**	**Notes**
Wearable device uptake	θ	4%	We defined uptake as the percent of the population that owns a wearable device, has downloaded the detection/notification application, *and* uses the device enough to collect sufficient data for detection. We estimated uptake would range from 0.5% to 7.5% at baseline. Tables C and D provide an example of this calculation.<br>Device ownership: Estimates from 2018 place device ownership in Canada between 22% and 25% [12,13].<br>Download rate: A study found that the baseline download rate of Germany's national contact tracing application was between ~8% and ~11%; incentives could increase this rate [14]. Paré and colleagues found that 57% of owners regularly track their health with their device [12]. Here, we looked at download rates ranging from 10% to 60%.<br>Utilization: In research studies where wearable devices were used to track health, usage rates ranged from as low as 24%, to 50% in the long run [15,16].
Adherence to wearable device notification	ψ	50%	We defined adherence as the proportion of wearable device users that comply with recommended next steps upon notification of potential infection. At baseline, next steps include seeking a confirmatory lab-based test, quarantining while awaiting a result, and self-isolating until recovery if the result is positive. With antigen tests, compliant users also take a confirmatory rapid antigen test prior to seeking a lab-based test.<br>Adherence levels: In Israel, at least ~53% of antigen test kit results were reported (~613,000 reports out of ~1,150,000 kits taken home) [17,18]. In the UK, duration-adjusted adherence to self-isolation was 42.5% [19]. In Norway, up to 70% of those with a suspected diagnosis and up to 86% with a positive diagnostic test adhered to self-isolation [20]. However, adherence could be as low as ~14%, if one considers Canada's COVID Alert contact tracing app's reporting rate in light of confirmed cases as of July 27, 2021 [21].<br>During Monte Carlo simulations, we modeled adherence as a beta random variable with a mean of 0.5 and a sample size of 1723; 1723 was the sample size for the relevant experiment in the Norway study.
Detection algorithm sensitivity	σ_w	80%	We defined sensitivity as the proportion of infected yet asymptomatic wearable device users (i.e., *Exposed*, *Presymptomatic*, and *Asymptomatic*) who receive a notification of potential infection prior to recovering and entering the *Removed* compartment. Alavi and colleagues' NightSignal algorithm achieved a sensitivity of ~80%, which is a plausible value based on other efforts to develop similar algorithms [22,23].<br>During Monte Carlo simulations, we modeled sensitivity as a beta random variable with a mean of 0.80 and a sample size of 84; 84 was the size of the sample used to calculate the NightSignal algorithm's sensitivity.
Detection algorithm specificity	ν_w	92%	We defined specificity as the probability that, on a given day, a healthy (i.e., *Susceptible*) user does not receive a notification of potential infection. Alavi and colleagues' NightSignal algorithm gave potentially healthy users 0.0819 false positive notifications ("red alerts") per day on average, corresponding to a specificity of ~92% [22].

			During Monte Carlo simulations, we modeled specificity as a beta random variable with a mean of 0.92 and a sample size of 818; alarm data from 818 potentially healthy users in Alavi and colleagues' dataset were used to calculate the false positive rate.
Detection algorithm sensitivity adjustment factor	κ	$(1/\alpha + 1/\tau + 1/\lambda)^{-1}$ days^{-1}	We assumed that σ_w is applied uniformly across *Exposed*, *Presymptomatic*, and *Asymptomatic* states such that by the time infectiousness ends, σ_w of infected users without symptoms are notified.
Detection algorithm specificity adjustment factor	χ	$(1)^{-1}$ days^{-1}	We assumed that ν_w is applied over a period of one day such that on any given day, *Susceptible* users receive an incorrect (i.e., false positive) notification of potential infection with probability $(1-\nu_w)$.
Rapid antigen test sensitivity	σ_a	91.1%	We used the lowest performance reported for Abbot's Panbio test [24]. Independent evaluations of this test suggest lower sensitivity in presymptomatic and asymptomatic individuals [25,26]. However, we reasoned that the receipt of a wearable-informed notification of potential infection raised the pretest probability of infection, thereby improving the negative predictive value (NPV) of the tests [27]. We performed a sensitivity analysis on the sensitivity of rapid antigen tests (Fig F).
Rapid antigen test specificity	ν_a	99.7%	We used the lowest performance reported for Abbot's Panbio test [24]. Independent evaluations suggest that this test's specificity is even higher [25,26].
Lab-based test turnaround time	ε	2 days	In general, 1-3 days based on Health Canada reporting [28].
Relative contribution to transmission	*a*	1	Used to study scenarios in which device users who do not receive a positive notification or who ignore a positive notification act in a riskier or more cautious fashion, respectively [29]. See Equation (2).

Model equations, in addition to Equation (1), used to extract transmission rate (β) from the IHME infection model:

$$\frac{dS}{dt} = -\pi \tag{3}$$

$$\frac{dE}{dt} = \pi - \alpha E \tag{4}$$

$$\frac{dI_p}{dt} = \alpha E - \tau I_p \tag{5}$$

$$\frac{dI_a}{dt} = \rho \tau I_p - \gamma I_a \tag{6}$$

$$\frac{dI_s}{dt} = (1-\rho)\tau I_p - \gamma I_s \tag{7}$$

$$\frac{dR}{dt} = \gamma I_a + \gamma I_s \tag{8}$$

Model equations, in addition to Equation (2), used to run simulations:

$$\frac{dS_w}{dt} = -\theta\pi - \psi\chi(1-\nu_w)(1-\nu_a)S_w + \frac{Q_i}{\epsilon} \tag{9}$$

$$\frac{dE_w}{dt} = \theta\pi - \psi\kappa\sigma_w\sigma_a E_w - \alpha E_w \tag{10}$$

$$\frac{dI_{p,w}}{dt} = \alpha E_w - \psi\kappa\sigma_w\sigma_a I_{p,w} - \tau I_{p,w} \tag{11}$$

$$\frac{dI_{a,w}}{dt} = \rho\tau I_{p,w} - \psi\kappa\sigma_w\sigma_a I_{a,w} - \gamma I_{a,w} \tag{12}$$

$$\frac{dI_{s,w}}{dt} = (1-\rho)\tau I_{p,w} - \gamma I_{s,w} \tag{13}$$

$$\frac{dS_{nw}}{dt} = -(1-\theta)\pi \tag{14}$$

$$\frac{dE_{nw}}{dt} = (1-\theta)\pi - \alpha E_{nw} \tag{15}$$

$$\frac{dI_{p,nw}}{dt} = \alpha E_{nw} - \tau I_{p,nw} \tag{16}$$

$$\frac{dI_{a,nw}}{dt} = \rho\tau I_{p,nw} - \gamma I_{a,nw} \tag{17}$$

$$\frac{dI_{s,nw}}{dt} = (1-\rho)\tau I_{p,nw} - \gamma I_{s,nw} \tag{18}$$

$$\frac{dR}{dt} = \gamma(I_{a,w} + I_{s,w} + I_{a,nw} + I_{s,nw}) + \frac{Q_c}{\epsilon} \tag{19}$$

$$\frac{dQ_i}{dt} = \psi\chi(1-\nu_w)(1-\nu_a)S_w - \frac{Q_i}{\epsilon} \tag{20}$$

$$\frac{dQ_c}{dt} = \psi\kappa\sigma_w\sigma_a(E_w + I_{p,w} + I_{a,w}) - \frac{Q_c}{\epsilon} \tag{21}$$

2. Estimating ranges for uptake

Below, Tables C and D delineate how we estimated plausible ranges for uptake in a baseline scenario. First, we multiplied the download rate by the proportion of the population that owns a device to obtain a plausible range for the proportion of the population that owns the application (Table C). Then, we calculated a range for uptake by multiplying the proportion of individuals that own the application by expected levels of utilization (Table D). Accounting for utilization was a necessary step because not all individuals who download the application to their device use it enough to provide sufficient data for the algorithm to function correctly [22,30].

We concluded that in a baseline scenario, uptake would likely range from 0.5% to 7.5%. Our assumptions around values for device ownership, the download rate, and utilization are listed in Table B above.

Table C: Calculation of the proportion of individuals who own the application.

**Application Ownership**	*Device Ownership*		
Download Rate	*22.0%*	*23.5%*	*25.0%*
10.0%	2.2%	2.4%	2.5%
35.0%	7.7%	8.2%	8.8%
60.0%	13.2%	14.1%	15.0%

Table D: Calculation of the proportion of individuals who own and use the application.

**Uptake**	*Utilization*		
Application Ownership	*24.0%*	*37.0%*	*50.0%*
2.2%	0.5%	0.8%	1.1%
8.6%	2.1%	3.2%	4.3%
15.0%	3.6%	5.6%	7.5%

3. Additional results

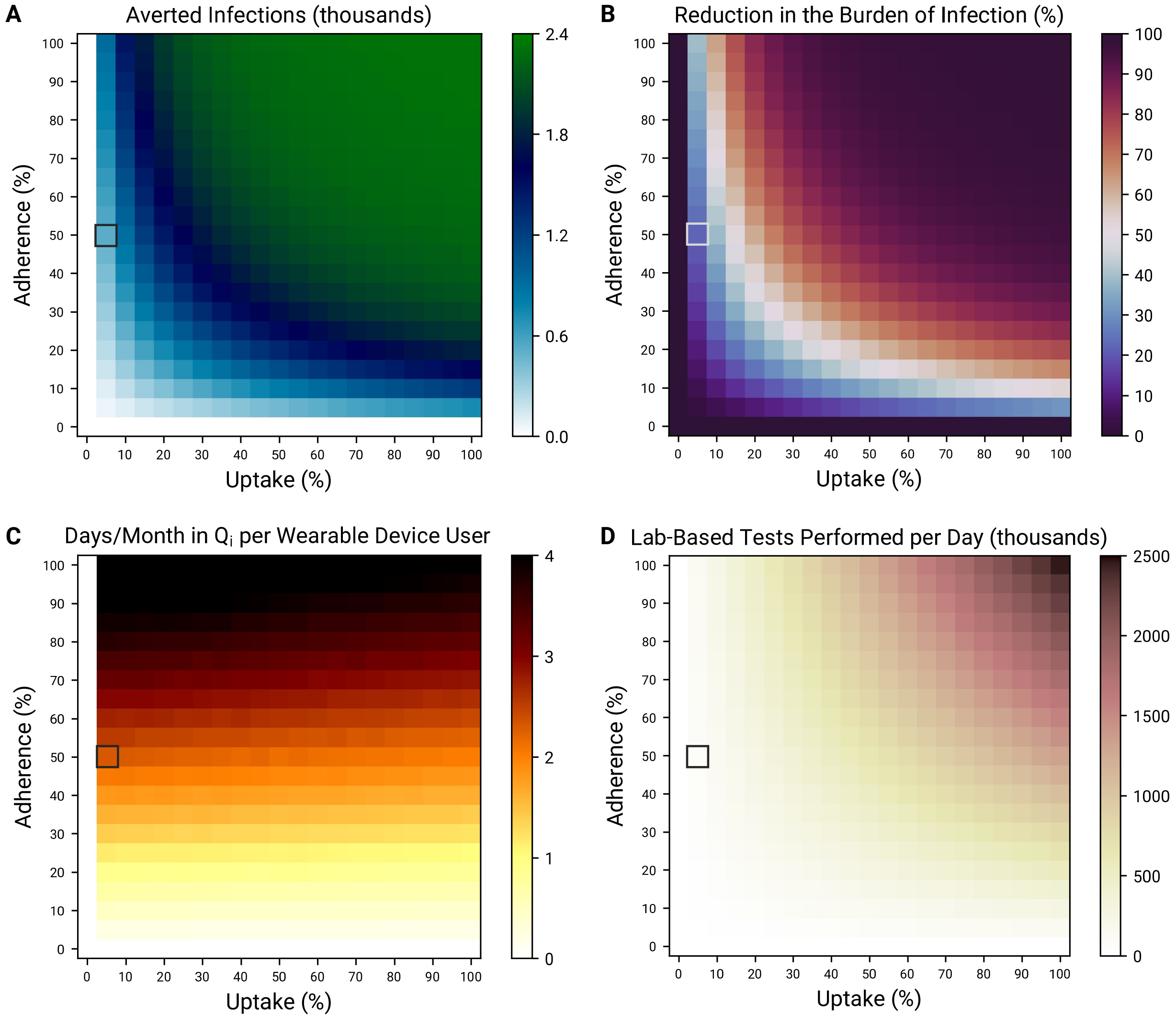

Fig A: Impact of simultaneously increasing uptake and adherence. Averted infections (A), reduction in the burden of infection (B), days incorrectly spent in quarantine per month per user (C), and average daily demand for lab-based tests (D), all over the simulation period, as a function of uptake and adherence. Grey boxes denote nominal sensitivity (80%) and specificity (92%).

Table E: Wearable sensor deployment scenarios under different technology and behavioural assumptions. 95% confidence intervals are listed in parentheses. This table serves as a counterpart to Table 1 so that analogous scenarios with and without confirmatory antigen tests can be compared.

Uptake (%)	Adherence (%)	Averted Infections (thousands)	Reduction in Burden of Infection (%)	Days/Month in Q_i per User (thousands)	Additional Lab-Based Tests Performed per Day
Nominal Detection Sensitivity (80%) and Specificity (92%) Scenario					
0.5	14	15.5 (13.3–17.8)	0.7 (0.6–0.8)	0.63 (0.48–0.79)	1.9 (1.5 – 2.4)
0.5	50	48.8 (44.1–53.3)	2.1 (1.9–2.3)	2.11 (1.66–2.60)	6.4 (5.1 – 7.9)
0.5	86	75.3 (69.2–81.1)	3.2 (3.0–3.5)	3.45 (2.77–4.19)	10.5 (8.4 – 12.7)
4.0	14	121.7 (104.4–139.5)	5.2 (4.5–6.0)	0.63 (0.48–0.80)	15.3 (11.6 – 19.3)
4.0	50	366.4 (333.9–398.7)	15.6 (14.2–17.0)	2.13 (1.68–2.61)	51.6 (40.7 – 63.2)
4.0	86	543.4 (503.7–579.4)	23.2 (21.5–24.7)	3.47 (2.78–4.22)	84.2 (67.5 – 102.4)
7.5	14	222.9 (193.3–253.4)	9.5 (8.2–10.8)	0.63 (0.48–0.79)	28.5 (21.7 – 36.1)
7.5	50	642.6 (589.3–694.7)	27.4 (25.1–29.6)	2.13 (1.68–2.62)	96.9 (76.6 – 119.0)
7.5	86	919.4 (859.9–974.4)	39.2 (36.7–41.6)	3.49 (2.79–4.22)	158.8 (126.8 – 192.1)
High Detection Sensitivity (96.0%) and Specificity (98.4%) Scenario					
0.5	14	14.9 (13.1–16.7)	0.6 (0.6–0.7)	0.13 (0.07–0.21)	0.4 (0.2 – 0.6)
0.5	50	45.5 (42.7–48.1)	1.9 (1.8–2.1)	0.45 (0.24–0.72)	1.4 (0.8 – 2.2)
0.5	86	68.0 (65.0–71.0)	2.9 (2.8–3.0)	0.76 (0.42–1.19)	2.3 (1.3 – 3.6)
4.0	14	117.1 (103.9–131.1)	5.0 (4.4–5.6)	0.13 (0.07–0.21)	3.1 (1.7 – 5.1)
4.0	50	342.7 (323.7–361.6)	14.6 (13.8–15.4)	0.45 (0.25–0.72)	11.1 (6.1 – 17.5)
4.0	86	497.1 (476.6–517.3)	21.2 (20.3–22.1)	0.77 (0.42–1.21)	18.8 (10.4 – 29.6)
7.5	14	215.6 (191.5–240.7)	9.2 (8.2–10.3)	0.13 (0.07–0.21)	5.9 (3.2 – 9.5)
7.5	50	604.7 (572.4–635.0)	25.8 (24.4–27.1)	0.45 (0.24–0.72)	20.8 (11.3 – 33.0)
7.5	86	850.7 (818.7–881.8)	36.3 (34.9–37.6)	0.77 (0.43–1.21)	35.3 (19.6 – 55.4)

4. Sensitivity analyses

First, to ensure our findings were robust to the underlying infection model, we replicated core analyses using estimates of π from the Imperial College London (ICL) infection model (Fig B) [31]. ICL and IHME estimates of π expectedly differ greatly, however, we confirmed that the relative public health impact of wearable sensor deployment remained consistent. In a baseline scenario (4% uptake, 50% adherence, 80% detection sensitivity, 92% detection specificity), we observed a 15.6% (95% CI: 14.2–16.9%) reduction in the burden of infection using IHME estimates of π. Here, with ICL estimates, we observed a 15.8% (95% CI: 14.5–17.2%) reduction. In both cases, between ~75,000 and ~125,000 device users were incorrectly quarantining on any given day and between ~40,000 and ~65,000 additional lab-based tests were required each day.

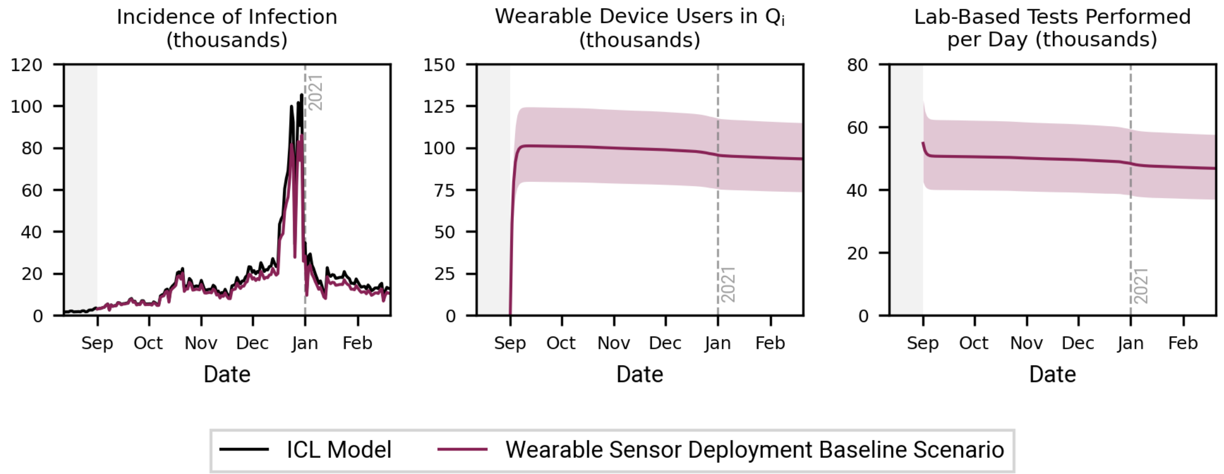

Fig B: Baseline scenario for wearable sensor deployment using Imperial College London's infection model. Time series depiction of the incidence of infection (A), the number of wearable device users incorrectly in quarantine (B), and the daily demand for lab-based tests (C). Uptake, adherence, detection sensitivity, and detection specificity are set to 4%, 50%, 80%, and 92%, respectively.

Second, we considered scenarios in which (1) the relative contribution to transmission of users who do not receive a positive notification increases due to a sense of false confidence, and (2) that of users who ignore a positive notification decreases due to a sense of caution (i.e., partial adherence; Fig C) [29]. Users stay in *Susceptible*, *Presymptomatic Infectious*, and *Asymptomatic Infectious* compartments either because they do not receive a positive notification or because they ignore a positive notification. By modulating a in Equation (2), we captured the weighted average change in relative contribution to transmission driven by these two groups. When a is below 1, the average user in these compartments acts more cautiously relative to historical behavior; when a is above 1, the average user acts in a riskier fashion. Increases in transmission among users relative to historical levels resulted in smaller reductions in the burden of infection while decreases had the opposite effect. The number of incorrect quarantines was not impacted. The implication of this finding is that public health leaders would need to communicate the limitations of wearable sensors with respect to detecting infections and emphasize that a lack of a notification does not rule out potential infection.

Third, we looked at the impact of asymptomatic prevalence (Fig D). It was important to perform this analysis because our model only accounts for notifications sent to presymptomatic and asymptomatic individuals, yet there is a lack of consensus on a specific value for the asymptomatic prevalence [6]. As expected, with greater asymptomatic prevalence, more individuals could benefit from wearable device use and more infections could be averted. The number of incorrect quarantines was not impacted.

Fourth, we investigated how the decrease in the number of *Susceptible* individuals resulting from incorrect quarantines influenced the number of averted infections in a baseline scenario (Fig E). We compared the number of averted infections with nominal (92%) and perfect (100%) detection specificity as detection sensitivity increased. In a baseline scenario, a meaningful proportion – 22.7% (95% CI: 13.1–32.5%) in the case of nominal detection sensitivity – of averted infections were driven by incorrect quarantines. This proportion decreased with increasing sensitivity. This

finding suggests that a substantial number of infections can be achieved even while attempting to minimize unnecessary quarantines, especially if detection sensitivity can be improved in parallel.

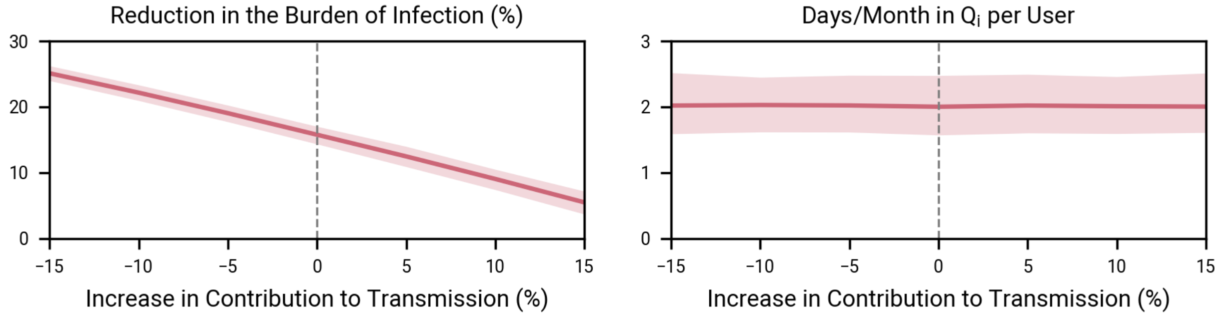

Fig C: Impact of decreased contribution to transmission among users who ignore a positive notification and of increased contribution among users not notified of potential infection in a baseline scenario. When a is below 1, the average wearable device user in the *Susceptible*, *Presymptomatic Infectious*, and *Asymptomatic Infectious* compartments acts more cautiously relative to historical behavior; when a is above 1, the average user in these groups acts in a riskier fashion. We assumed 4% uptake, 50% adherence, and that transmission among non-users was unchanged. The vertical dashed grey line at 0% reflects the change in contribution when a is nominally set to 1.

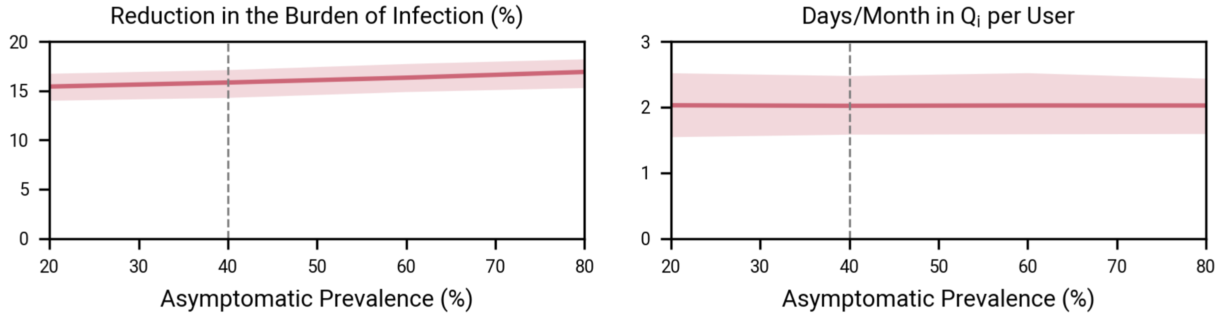

Fig D: Impact of asymptomatic prevalence in a baseline scenario. We assumed 4% uptake and 50% adherence. We continued to model asymptomatic prevalence as a beta-distributed random variable (Table A). The vertical dashed grey line represents nominal asymptomatic prevalence (40%).

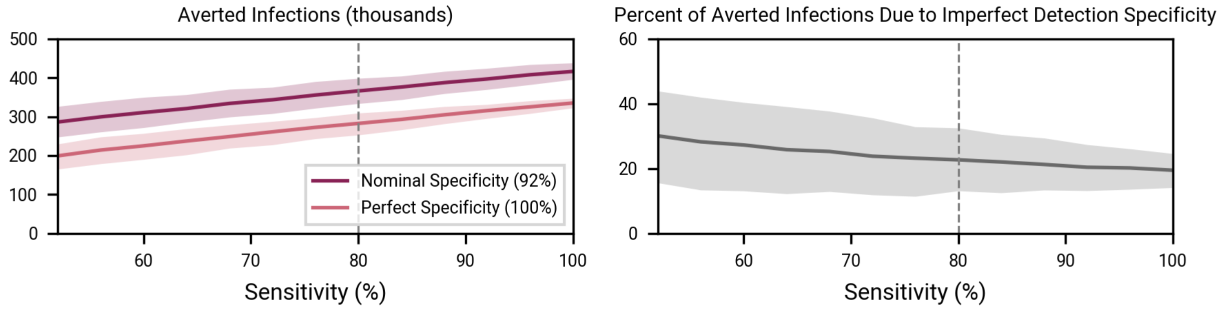

Fig E: Impact of incorrect quarantines on averted infections in a baseline scenario. The percentages in the plot on the right are calculated by dividing the difference in the number of averted infections with nominal and perfect detection specificity by the number of averted infections with nominal detection specificity. We assumed 4% uptake and 50% adherence. The vertical dashed grey line depicts nominal detection algorithm sensitivity (80%).

Fifth, we investigated the impact of lower rapid antigen test sensitivity and made two observations (Fig F). First, fewer infections were averted when rapid antigen tests were used at all – as discussed, using antigen tests to minimize incorrect quarantines increased the pool of *Susceptible* individuals. Second, averted infections grew linearly with test

sensitivity: a ~10% increase in test sensitivity resulted in a ~1% reduction in the burden of infection. These two effects result in a tradeoff between missing more *Infectious* individuals (imperfect antigen test sensitivity) and decreasing false positive prompts to seek a lab-based test and quarantine while waiting for the results (near perfect antigen test specificity). We believe the use of antigen tests as a complementary mechanism could be justified. Although fewer infections are averted relative to wearable sensor deployment without antigen tests, hundreds of thousands of infections are still averted relative to the counterfactual scenario – and in a resource-efficient and socially acceptable fashion. Certainly, infected individuals with a false negative antigen test might act in a riskier fashion – even still, there remains an opportunity to avert a substantial number of infections (Fig C). We also point out that improving detecting algorithm sensitivity could help counteract the effect of imperfect antigen test sensitivity (Table 1).

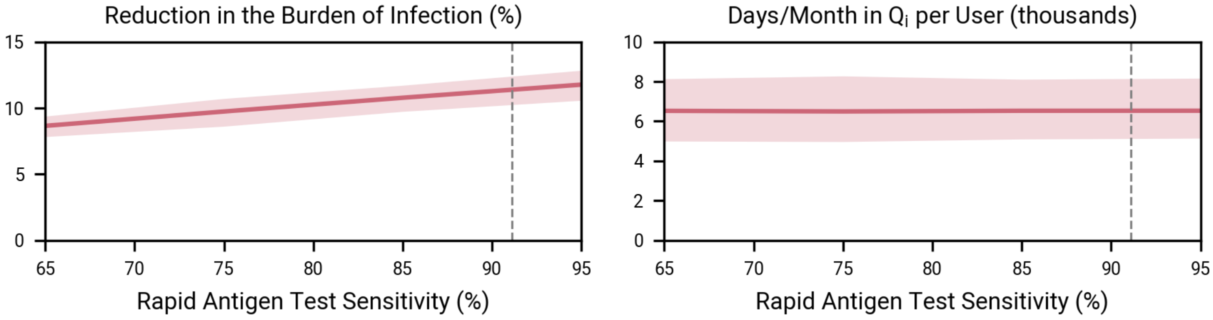

Fig F: Impact of antigen test sensitivity. We assumed 4% uptake and 50% adherence. The vertical dashed grey line represents nominal antigen test sensitivity (91.7%).

Sixth, we explored the impact of lab-based test turnaround time to determine whether minimizing this variable should be a policy priority (Fig G). Throughout this study, we set turnaround time to its nominal value of two days. With longer turnaround times, individuals incorrectly in quarantine would remain there longer, further decreasing the pool of *Susceptible* individuals. As expected, a greater reduction in the burden of infection occurs. However, there are other mechanisms (e.g., improving detection sensitivity) for increasing averted infections that are not accompanied by as large of an increase to social costs. Thus, as is already the case outside the context of wearable sensor deployment, it would make sense to minimize turnaround time.

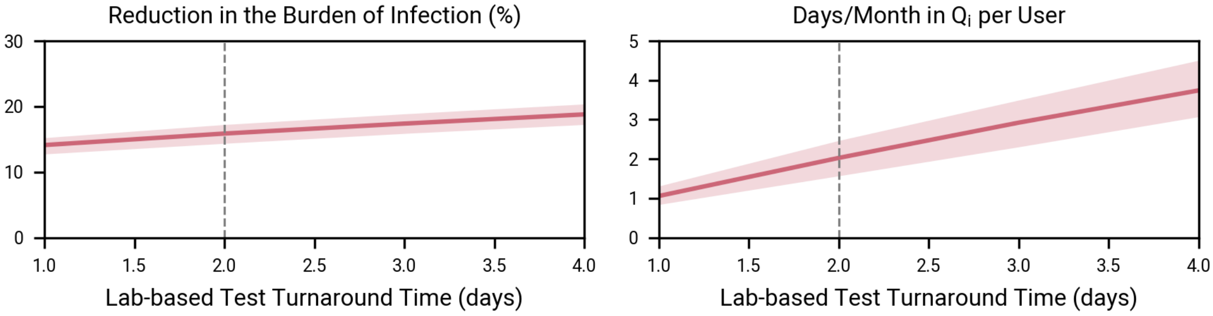

Fig G: Impact of lab-based test turnaround time. We assumed 4% uptake and 50% adherence. The vertical dashed grey line represents nominal lab-based test turnaround time (2 days).

Finally, we considered how the achievable reduction in the burden of infection might vary as virus transmissibility increases (Fig H). Changes in transmissibility can be driven by several factors. For example, unvaccinated individuals infected with the Delta variant had higher viral loads relative those infected with the SARS-CoV-2 ancestral strain [32]. However, Delta variant infections elicited lower viral loads in recently vaccinated individuals as compared to unvaccinated individuals [32,33]. Further, an increase in the degree of immune evasion as opposed to an increase in viral load may drive the increased transmissibility of the Omicron variant [32,34]. We used the same compartmental framework (Fig 1) to explore simple hypothetical scenarios with changes in transmissibility (Fig H). We simulated an epidemic in a population with 10 million individuals (no prior immunity) and explored the effect of varying a constant transmission rate (β) from 2 (0% increase in relative transmissibility) to 3 (50% increase in relative transmissibility)

infections per infectious individual per day. We measured the reduction in the burden of infection achieved with baseline wearable sensor deployment assumptions (4% uptake, 50% adherence, 80% sensitivity, 92% specificity). While this analysis does not capture temporal changes in transmissibility that may be associated with factors such as the emergence of variants of concern, our findings confirm the intuition that fewer infections would been averted with greater viral transmissibility. This intuition is in agreement with qualitative findings from other studies that have modeled pandemic mitigation strategies [35,36].

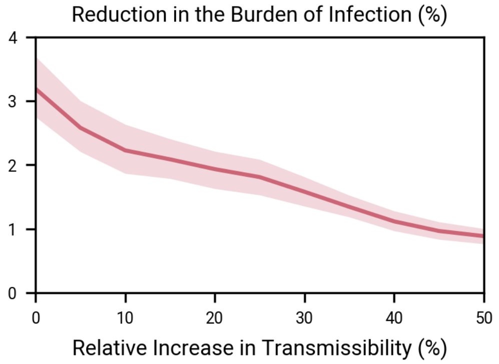

Fig H: Impact of increases in transmissibility in a hypothetical epidemic scenario. We used the same modeling framework as in Fig 1 to simulate an epidemic in a population with 10 million individuals (no prior immunity). We held transmission rate constant through each simulated epidemic, progressively increasing its value from 2 (baseline) to 3 (50% increase) infections per infectious individual per day. We used baseline wearable sensor deployment assumptions (4% uptake, 50% adherence, 80% sensitivity, 92% specificity).

5. References

1. Zhao S, Tang B, Musa SS, Ma S, Zhang J, Zeng M, et al. Estimating the generation interval and inferring the latent period of COVID-19 from the contact tracing data. Epidemics. 2021;36: 100482. doi:10.1016/j.epidem.2021.100482

2. Aleta A, Martín-Corral D, Pastore y Piontti A, Ajelli M, Litvinova M, Chinazzi M, et al. Modelling the impact of testing, contact tracing and household quarantine on second waves of COVID-19. Nat Hum Behav. 2020;4: 964–971. doi:10.1038/s41562-020-0931-9

3. Lau YC, Tsang TK, Kennedy-Shaffer L, Kahn R, Lau EHY, Chen D, et al. Joint Estimation of Generation Time and Incubation Period for Coronavirus Disease 2019. J Infect Dis. 2021; jiab424. doi:10.1093/infdis/jiab424

4. Xin H, Wong JY, Murphy C, Yeung A, Taslim Ali S, Wu P, et al. The Incubation Period Distribution of Coronavirus Disease 2019: A Systematic Review and Meta-analysis. Clin Infect Dis. 2021;73: 2344–2352. doi:10.1093/cid/ciab501

5. Tang X, Musa SS, Zhao S, Mei S, He D. Using Proper Mean Generation Intervals in Modeling of COVID-19. Front Public Health. 2021;9: 691262. doi:10.3389/fpubh.2021.691262

6. Oran DP, Topol EJ. Prevalence of Asymptomatic SARS-CoV-2 Infection: A Narrative Review. Ann Intern Med. 2020;173: 362–367. doi:10.7326/M20-3012

7. Zhao S, Department of Applied Mathematics, Hong Kong Polytechnic University, Hong Kong, China, School of Nursing, Hong Kong Polytechnic University, Hong Kong, China, JC School of Public Health and Primary Care, Chinese University of Hong Kong, Hong Kong, China, CUHK Shenzhen Research Institute, Shenzhen, China. Estimating the time interval between transmission generations when negative values occur in the serial interval data: using COVID-19 as an example. Math Biosci Eng. 2020;17: 3512–3519. doi:10.3934/mbe.2020198

8. Peirlinck M, Linka K, Sahli Costabal F, Kuhl E. Outbreak dynamics of COVID-19 in China and the United States. Biomech Model Mechanobiol. 2020;19: 2179–2193. doi:10.1007/s10237-020-01332-5

9. Li R, Pei S, Chen B, Song Y, Zhang T, Yang W, et al. Substantial undocumented infection facilitates the rapid dissemination of novel coronavirus (SARS-CoV-2). Science. 2020;368: 489–493. doi:10.1126/science.abb3221

10. He D, Zhao S, Lin Q, Zhuang Z, Cao P, Wang MH, et al. The relative transmissibility of asymptomatic COVID-19 infections among close contacts. Int J Infect Dis. 2020;94: 145–147. doi:10.1016/j.ijid.2020.04.034

11. Buitrago-Garcia D, Egli-Gany D, Counotte MJ, Hossmann S, Imeri H, Ipekci AM, et al. Occurrence and transmission potential of asymptomatic and presymptomatic SARS-CoV-2 infections: A living systematic review and meta-analysis. Ford N, editor. PLOS Med. 2020;17: e1003346. doi:10.1371/journal.pmed.1003346

12. Paré G, Leaver C, Bourget C. Diffusion of the Digital Health Self-Tracking Movement in Canada: Results of a National Survey. J Med Internet Res. 2018;20: e177. doi:10.2196/jmir.9388

13. Sujay Vailshery L. Activity fitness tracker household penetration rate in Canada in 2018, by province. Statista; 2021 Jan. Available: https://www.statista.com/statistics/1036752/canada-activity-fitness-tracker-ownership-rate-by-province/

14. Munzert S, Selb P, Gohdes A, Stoetzer LF, Lowe W. Tracking and promoting the usage of a COVID-19 contact tracing app. Nat Hum Behav. 2021;5: 247–255. doi:10.1038/s41562-020-01044-x

15. Silva de Lima AL, Hahn T, Evers LJW, de Vries NM, Cohen E, Afek M, et al. Feasibility of large-scale deployment of multiple wearable sensors in Parkinson's disease. Arias-Carrion O, editor. PLOS ONE. 2017;12: e0189161. doi:10.1371/journal.pone.0189161

16. Radin JM, Wineinger NE, Topol EJ, Steinhubl SR. Harnessing wearable device data to improve state-level real-time surveillance of influenza-like illness in the USA: a population-based study. Lancet Digit Health. 2020;2: e85–e93. doi:10.1016/S2589-7500(19)30222-5

17. Trabelsi Haddad T. Returning to school, again: "Operation Antigen 2" is underway, and only negatives will be admitted to institutions. ynet. 28 Sep 2021. Available: https://www.ynet.co.il/news/article/b1itlgbvy. Accessed 17 Dec 2021.

18. Trabelsi Haddad T. 613,000 reported, about 550 positive: Antigen results - and those that will arrive tomorrow without testing. ynet. 30 Sep 2021. Available: https://www.ynet.co.il/news/article/bjz3frm4t. Accessed 17 Dec 2021.

19. Smith LE, Potts HWW, Amlôt R, Fear NT, Michie S, Rubin GJ. Adherence to the test, trace, and isolate system in the UK: results from 37 nationally representative surveys. BMJ. 2021; n608. doi:10.1136/bmj.n608

20. Carlsen EØ, Caspersen IH, Trogstad L, Gjessing HK, Magnus P. Public adherence to governmental recommendations regarding quarantine and testing for COVID-19 in two Norwegian cohorts. Epidemiology; 2020 Dec. doi:10.1101/2020.12.18.20248405

21. Government of Canada. How many people are using COVID Alert. Available: https://www.canada.ca/en/public-health/services/diseases/coronavirus-disease-covid-19/covid-alert.html#a6

22. Alavi A, Bogu GK, Wang M, Rangan ES, Brooks AW, Wang Q, et al. Real-time alerting system for COVID-19 and other stress events using wearable data. Nat Med. 2021 [cited 15 Dec 2021]. doi:10.1038/s41591-021-01593-2

23. Gadaleta M, Radin JM, Baca-Motes K, Ramos E, Kheterpal V, Topol EJ, et al. Passive detection of COVID-19 with wearable sensors and explainable machine learning algorithms. Npj Digit Med. 2021;4: 166. doi:10.1038/s41746-021-00533-1

24. Abbot. In vitro diagnostic rapid test for qualitative detection of SARS-CoV-2 antigen (Ag). Abbot; Available: https://www.globalpointofcare.abbott/en/product-details/panbio-covid-19-ag-antigen-test.html

25. Torres I, Poujois S, Albert E, Colomina J, Navarro D. Evaluation of a rapid antigen test (Panbio™ COVID-19 Ag rapid test device) for SARS-CoV-2 detection in asymptomatic close contacts of COVID-19 patients. Clin Microbiol Infect. 2021;27: 636.e1-636.e4. doi:10.1016/j.cmi.2020.12.022

26. Winkel B, Schram E, Gremmels H, Debast S, Schuurman R, Wensing A, et al. Screening for SARS-CoV-2 infection in asymptomatic individuals using the Panbio COVID-19 antigen rapid test (Abbott) compared with RT-PCR: a prospective cohort study. BMJ Open. 2021;11: e048206. doi:10.1136/bmjopen-2020-048206

27. Woloshin S, Patel N, Kesselheim AS. False Negative Tests for SARS-CoV-2 Infection — Challenges and Implications. N Engl J Med. 2020;383: e38. doi:10.1056/NEJMp2015897

28. Government of Canada. Testing for COVID-19: How and where we test for active infections. 2021 Dec. Available: https://www.canada.ca/en/public-health/services/diseases/2019-novel-coronavirus-infection/symptoms/testing/diagnosing.html

29. Cleary JL, Fang Y, Sen S, Wu Z. A Caveat to Using Wearable Sensor Data for COVID-19 Detection: The Role of Behavioral Change after Receipt of Test Results. Infectious Diseases (except HIV/AIDS); 2021 Apr. doi:10.1101/2021.04.17.21255513

30. Dunn J, Shandhi MMH, Cho P, Roghanizad A, Singh K, Wang W, et al. A Method for Intelligent Allocation of Diagnostic Testing by Leveraging Data from Commercial Wearable Devices: A Case Study on COVID-19. In Review; 2022 Apr. doi:10.21203/rs.3.rs-1490524/v1

31. Walker PGT, Whittaker C, Watson OJ, Baguelin M, Winskill P, Hamlet A, et al. The impact of COVID-19 and strategies for mitigation and suppression in low- and middle-income countries. Science. 2020;369: 413–422. doi:10.1126/science.abc0035

32. Puhach O, Adea K, Hulo N, Sattonnet P, Genecand C, Iten A, et al. Infectious viral load in unvaccinated and vaccinated individuals infected with ancestral, Delta or Omicron SARS-CoV-2. Nat Med. 2022 [cited 12 Jul 2022]. doi:10.1038/s41591-022-01816-0

33. Levine-Tiefenbrun M, Yelin I, Alapi H, Katz R, Herzel E, Kuint J, et al. Viral loads of Delta-variant SARS-CoV-2 breakthrough infections after vaccination and booster with BNT162b2. Nat Med. 2021;27: 2108–2110. doi:10.1038/s41591-021-01575-4

34. Kozlov M. How does Omicron spread so fast? A high viral load isn't the answer. Nature. 2022; d41586-022-00129-z. doi:10.1038/d41586-022-00129-z

35. Chin ET, Huynh BQ, Chapman LAC, Murrill M, Basu S, Lo NC. Frequency of Routine Testing for Coronavirus Disease 2019 (COVID-19) in High-risk Healthcare Environments to Reduce Outbreaks. Clin Infect Dis. 2021;73: e3127–e3129. doi:10.1093/cid/ciaa1383

36. Grantz KH, Lee EC, D'Agostino McGowan L, Lee KH, Metcalf CJE, Gurley ES, et al. Maximizing and evaluating the impact of test-trace-isolate programs: A modeling study. PLOS Med. 2021;18: e1003585. doi:10.1371/journal.pmed.1003585
